# Signal of carbon exchange in Qilian mountain based on eddy covariance and remote sensing data

**DOI:** 10.1016/j.isci.2026.116949

**Published:** 2026-07-27

**Authors:** Chuntan Han, Yiwen Liu, Xiaobo Wang, Zhangwen Liu, Guohua Liu, Rensheng Chen

**Affiliations:** 1 State Key Laboratory of Ecological Safety and Sustainable Development in Arid Lands, Northwest Institute of Eco-Environment and Resources, Chinese Academy of Sciences, Lanzhou, China; 2 The Core Facilities and Services, Northwest Institute of Eco-Environment and Resources, Chinese Academy of Sciences, Lanzhou, China; 3 College of Geography and Tourism, Hengyang Normal University, Hengyang, China; 4 University of Chinese Academy of Sciences, Beijing, China

**Keywords:** Qinghai-Tibet plateau, carbon sink, net ecosystem productivity, eddy covariance, alpine ecosystem

## Abstract

Climate change threatens carbon sequestration on the Qinghai-Tibet plateau, particularly in the Qilian mountains on its northeastern edge. We combined eddy covariance measurements from five sites at altitudes of 3,000–4,200 m spanning 2011 to 2022 with remote sensing data to evaluate carbon sink dynamics. Mean net ecosystem productivity (NEP) from eddy data were 311.2 g C m^−2^ year^−1^, compared with 185.3 g C m^−2^ year^−1^ from remote sensing. NEP trends varied with altitude. Above roughly 3,700 m NEP may increase, whereas lower elevations show mixed signals. Overall, gross primary productivity increased faster than ecosystem respiration, producing a net NEP rise of 8.6 ± 4.3 g C m^−2^ year^−2^. Upscaled tower estimates indicate a regional carbon uptake of 6.25 × 10^10^ kg C year^−1^, while remote sensing estimates give 3.72 × 10^10^ kg C year^−1^. This substantial carbon sink could significantly lower the cost of achieving carbon neutrality.

## Highlights

• Eddy measurement reveals an effective carbon sink in the Qilian mountains
• GPP growth surpasses respiration, driving net carbon gain
• Regional carbon sink supports carbon neutrality goals

## Graphical abstract

**Figure:**
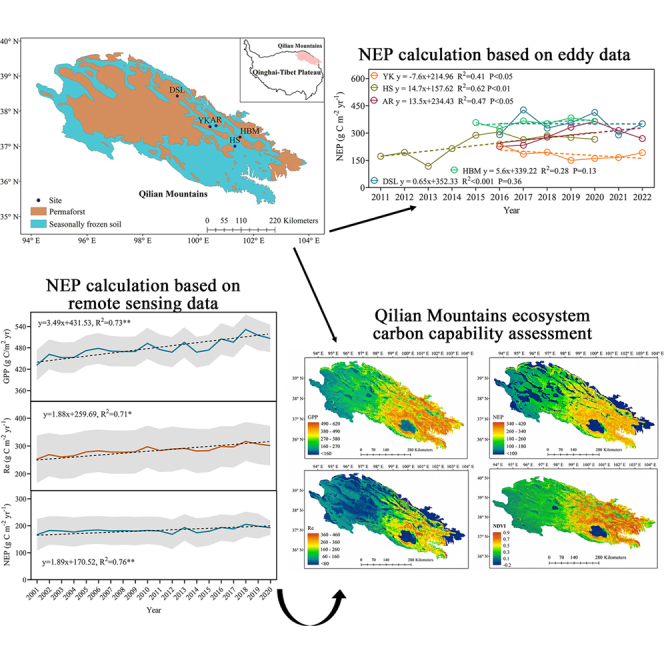

## Teaser

Earth-surface processes; Geomatics; Remote sensing

## Introduction

Terrestrial ecosystems serve as a vital carbon sink, playing a crucial role in the global carbon cycle. In recent years, the interaction between global warming and rising atmospheric greenhouse gas levels has attracted widespread attention regarding carbon storage in terrestrial ecosystems.^1^^,^^2^ In high-altitude regions, permafrost covers 25% of the land area in the northern hemisphere and stores approximately 1300 Pg C.^3^ Studies have indicated that the impact of warming in high-altitude regions is characterized by a thickening of the active layer, increased soil temperatures, and permafrost degradation.^4^ These factors undoubtedly contribute directly to carbon loss and may trigger carbon-climate feedback loops.^5^^,^^6^

The Qinghai-Tibet plateau (QTP), often referred to as the “third pole” of the Earth, serves as a significant carbon sink, absorbing and storing substantial amounts of CO_2_.^7^ This feature plays a critical role in the terrestrial carbon cycle and in mitigating climate change. The QTP contains the largest expanse of permafrost in mid- to low-latitude regions, covering 1.06 × 10^6^ km^2^^,^^8^ with an estimated 160 Pg C of carbon stored, representing 8.7% of the total in the northern hemisphere.^9^^,^^10^ However, the QTP is undergoing continuous warming at twice the global average rate of 0.12°C per decade,^11^^,^^12^ which may lead to the release of significant amounts of carbon,^3^ potentially triggering positive feedback to warming and threatening its carbon sink function,^13^ thereby increasing the cost of achieving carbon neutrality.^14^ However, this conclusion remains uncertain.^15^^,^^16^^,^^17^ The uncertainty primarily stems from an unclear balance between the enhancement of carbon uptake (e.g., vegetation greening and extended growing season) and the acceleration of carbon release (e.g., permafrost carbon thawing and the temperature sensitivity of respiration).

The northeastern edge of the QTP borders the Loess plateau to the east, which is a characteristic vegetation-desert ecotone and serves as a vital ecological barrier in western China, playing a crucial role in regulating climate patterns and supporting biodiversity. Furthermore, the northeastern edge of the QTP is influenced by westerlies, the plateau monsoon, and the east Asian monsoon, generating thermal low-pressure systems that accelerate the degradation of permafrost more rapidly and significantly.^1^^,^^18^ Against this backdrop, it is crucial to explore whether carbon emissions in this region have intensified and to examine the trends in carbon sink dynamics. Additionally, addressing these issues will aid in understanding potential carbon sequestration, which is equally important for formulating strategies to enhance carbon storage and mitigate climate change in the northeastern edge of the QTP.^19^^,^^20^

**Figure 1:**
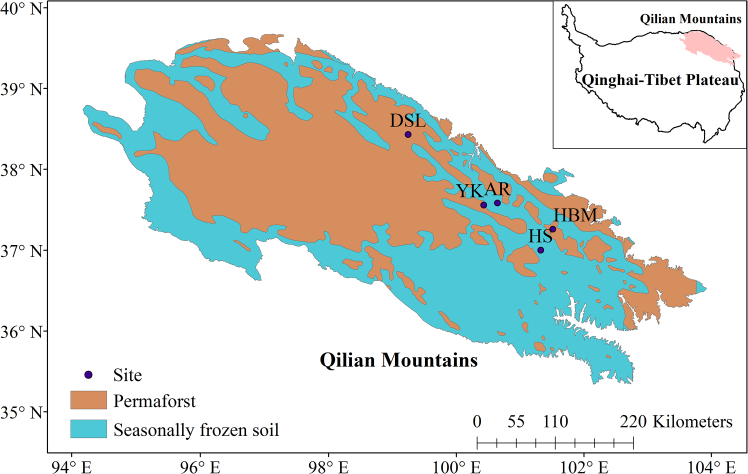
Location of the OPEC sites on the northeastern edge of the QTP and distribution of permafrost The scale bars represent 220 km in the figure.

Therefore, the Qilian mountains (QLMs), located on the northeastern edge of the QTP, were selected as the study area (Figure 1). Utilizing a unique decade-long open-path eddy c covariance (OPEC) dataset from five sites (2011–2022) spanning meadows and shrubs from ∼3,000 to 4,200 m (Table 1), this study provides a novel, mechanistic understanding of carbon sink vulnerability and stability under climate change. It was assumed that warming and humidification would promote net carbon uptake more significantly than net carbon emission. Specifically, research aims to (1) quantity the altitudinal dependence of carbon sink capacity trends and short-term variability, testing the hypothesis that high-altitude ecosystem show both the strongest sink enhancement and the greatest susceptibility to environmental perturbations; (2) identify and rank the key environmental drivers controlling carbon fluxes along the gradient; and (3) assess the cross-scale consistency between ground-based open-path eddy covariance (OPEC) observations and remote sensing products, evaluating the reliability of satellite-derived carbon flux estimates in this complex terrain. This integrated approach offers a comprehensive perspective that is crucial for predicting the future of this fragile carbon sink under climate change.

**Table 1:** Detailed information of open-path eddy covariance sites

Site	Ecosystem	Lon.	Lat.	Alt.	H (m)	Dominant species	Time range
AR	Meadow	100.46	38.04	3,033	3.5	*Stipa capillata* L.	2016–2022
HBM	Meadow	101.38	37.75	3,250	2.2	*Carex tibetikobresia* S. R. Zhang	2015–2020
YK	Meadow	100.24	38.01	4,148	3.2	*Carex alatauensis* S. R. Zhang	2016–2022
DSL	Swamp meadow	98.94	38.84	3,739	4.5	*Carex tibetikobresia* S. R. Zhang	2016–2022
HS	Shrub	101.21	37.48	3,180	2.2	*Potentilla fruticosa* L.	2011–2020

Note, Lon. means longitude. Lat. means latitude. Alt. means altitude. H means the height of the open-path eddy-covariance.

## Results

### Trend characteristics of net ecosystem productivity during the observation period

**Figure 2:**
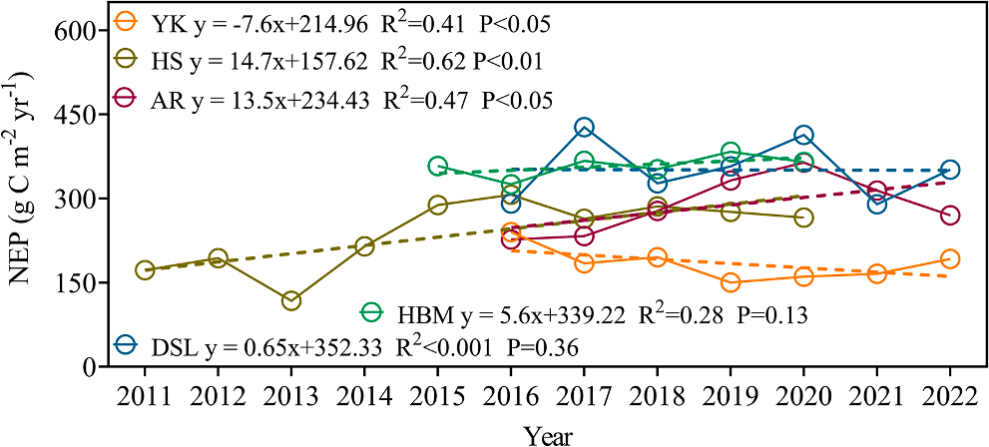
Observational trends of NEP in the northeast of the QTP at the annual scale The unit of change rate (slope) of the NEP time trend for different sites in the figure is g C m^−2^ year^−2^. Time series are from eddy covariance CO_2_ flux observations. Temporal trends were tested by ordinary least-squares linear regression, and *p* values are shown in the figure. *n* represents the number of annual site-level observations used in each regression (AR, *n* = 7; DSL, *n* = 7; HBM, *n* = 6; HS, *n* = 10; YK, *n* = 7). x in the equations in the figure is the sequence of years starting from the observation time, i.e., x = 1 for the first observation year, x = 2 for the second observation year, etc.

During the observation period, all sites consistently exhibited a net carbon sink across the years with mean net ecosystem productivity (NEP) of 311.2 ± 127.9 g C m^−2^ year^−1^ (Figure 2). Among these, the highest net CO_2_ sink was observed in swamp meadow (351.1 ± 54.3 g C m^−2^ year^−1^, DSL for 2016–2022), followed by meadow (273.1 ± 80.7 g C m^−2^ year^−1^, mean value including AR for 2016–2022, HBM for 2015–2020 and YK for 2016–2022), and shrub (238.6 ± 61.3 g C m^−2^ year^−1^, HS for 2011–2020). Additionally, three sites (DSL, YK, and HBM) were characterized by permafrost, with a mean NEP of 294.9 ± 91.0 g C m^−2^ year^−1^. On average, the annual growth rate of NEP in the study area was 8.6 ± 6.7 g C m^−2^ year^−2^ (*p* < 0.05). Among all sites, the fastest growth rate of NEP occurred at HS, with a rate of 14.7 g C m^−2^ year^−2^, while the smallest growth rate was observed at HBM, with a rate of 5.6 g C m^−2^ year^−2^. High values at AR (with a rate of 13.5 g C m^−2^ year^−2^) and HS affected the overall NEP growth rate (14.7 g C m^−2^ year^−2^). Ecosystem respiration also displayed an increasing trend (Figure S1), with an average growth rate of 7.5 g C m^−2^ year^−2^. The highest and lowest growth rates for Re were observed at HS (15.5 g C m^−2^ year^−2^) and HBM (1.6 g C m^−2^ year^−2^), respectively. NEP exhibited a fluctuating trend at DSL (with a rate of 0.65 g C m^−2^ year^−2^), whereas YK showed a decreasing trend during the observation period, with a rate of −7.6 g C m^−2^ year^−2^. Assuming that the NEP rates at DSL and YK remain unchanged, YK’s carbon sink pattern may reverse by the middle of this century, while the ecosystem at DSL would maintain a stable carbon surplus. However, starting from 2019, NEP at YK has displayed an increasing trend and continued to rise under the influence of warming.

**Figure 3:**
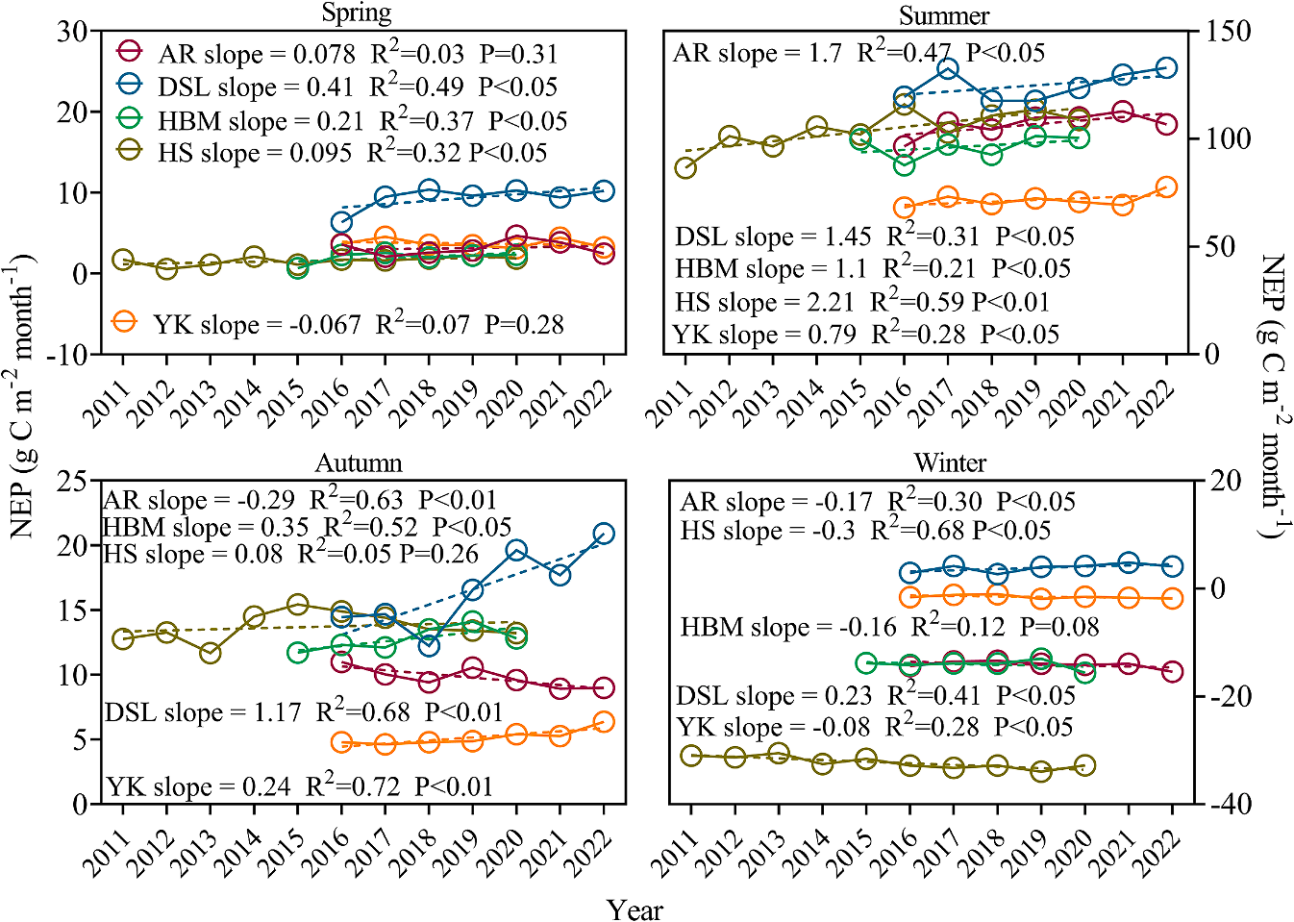
Observational trends of NEP in the northeast of the QTP at the seasonal scale The unit of seasonal change rate (slope) of NEP time trend for different sites in the figure is g C m^−2^ month^−1^ year^−1^. Time series are from eddy covariance CO_2_ flux observations. Temporal trends were tested by ordinary least-squares linear regression, and *p* values are shown in the figure. *n* represents the number of annual seasonal means used in each site-level regression (AR, *n* = 7; DSL, *n* = 7; HBM, *n* = 6; HS, *n* = 10; YK, *n* = 7). Spring was March–May, summer was June–August, autumn was September–November, and winter was December–February.

At the monthly scale, DSL consistently exhibited the highest NEP across seasons (Figure 3), with a mean NEP of 9.4 ± 1.4 g C m^−2^ month^−1^ and a rate of change of spring NEP over 2016 to 2022 of 0.41 g C m^−2^ month^−1^ year^−1^. No significant variations were observed among the other ecosystems during spring, with a mean NEP of 2.5 ± 1.1 g C m^−2^ month^−1^. This period contributed significantly to the annual NEP, and variations between different ecosystems began to emerge. The average rate of change in NEP at each site exhibited an increasing trend, with a mean rate of 1.6 ± 0.51 g C m^−2^ month^−1^ year^−1^. The sites with the fastest and slowest summer growth rates were HS (2.21 g C m^−2^ month^−1^ year^−1^) and YK (0.79 g C m^−2^ month^−1^ year^−1^), respectively.

The fluctuations and variations in NEP during autumn were also pronounced, marking a decline in carbon absorption capacity and the cessation of vegetation growth at the onset of the non-growing season. However, microbial decomposition of organic matter persisted, resulting in increased carbon emissions. Observational results revealed that NEP at DSL and YK exhibited a significant increasing trend during autumn, with mean rates of 1.17 g C m^−2^ month^−1^ year^−1^ and 0.24 g C m^−2^ month^−1^ year^−1^, respectively, while the mean rate at other sites was −0.14 g C m^−2^ month^−1^ year^−1^. The NEP trends across different ecosystems remained stable during winter. HS exhibited the highest carbon emissions during winter, averaging 32.3 ± 4.3 g C m^−2^ month^−1^, while DSL continued to function as a weak carbon sink, with an average NEP of 3.8 ± 0.7 g C m^−2^ month^−1^. At YK, the highest-altitude site, vegetation growth and microbial metabolic activities were severely constrained, and NEP indicated a weak carbon source (−1.5 ± 0.32 g C m^−2^ month^−1^) during winter. Additionally, there were no significant variations in carbon emissions between AR and HBM, with an average value of −14.1 ± 0.73 g C m^−2^ month^−1^.

### Characteristics of the net ecosystem productivity time series

**Figure 4:**
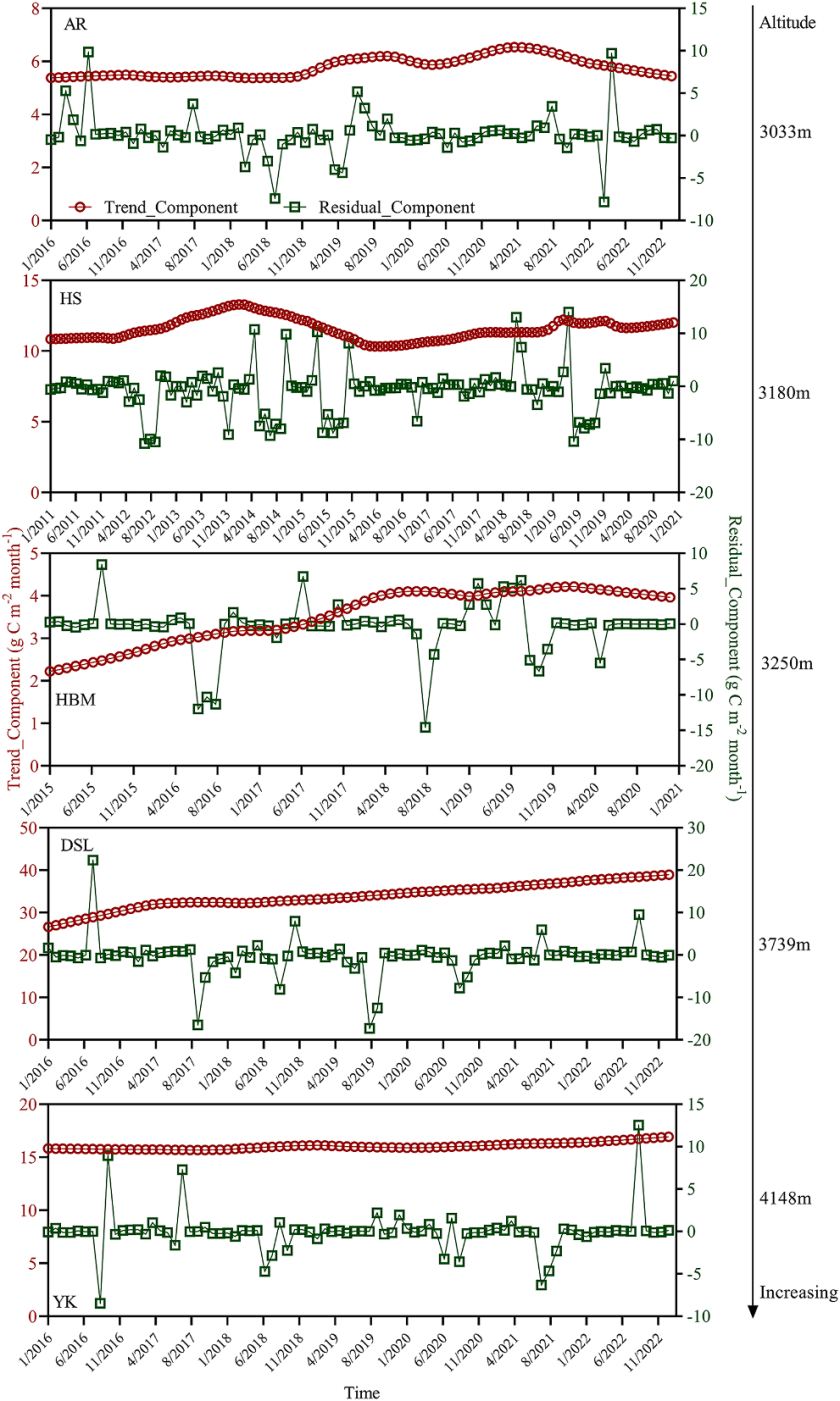
Trend and residual components of NEP time series obtained by seasonal-trend decomposition using Loess (STL) decomposition *n* represents the number of monthly aggregate observations used in the STL analysis (AR, *n* = 84; DSL, *n* = 84; HBM, *n* = 72; HS, *n* = 120; YK, *n* = 84).

Long-term increasing trends in NEP were consistently observed across different sites, with the magnitude of increasing exhibiting a pronounced altitude gradient (Figure 4). High-altitude ecosystems (YK: 4,148 m; DSL: 3,739 m) maintained the highest intensities of monthly NEP, with DSL demonstrating the most pronounced absolute growth (from 26.6 to 38.9 g C m^−2^ month ^−1^) and YK showing a high but relatively stable baseline (from 15.8 to 16.9 g C m^−2^ month ^−1^). Meanwhile mid-altitude sites (HBM: 3,250 m; HS: 3,180 m) showed moderate increases (HBM: 2.2 to 4.2 g C m^−2^ month ^−1^; 2015–2020), and the lowest-elevation site (AR: 3,033 m) exhibited the gentlest trend (5.4–6.5 g C m^−2^ month ^−1^). Short-term NEP variability, captured by residual components, revealed altitude-dependent responses to environmental changes (Figures 4 and S2). High-altitude sites displayed the widest residual fluctuations (YK: −8.5 to +12.5 g C m^−2^ month ^−1^; DSL: −17.3 to +22.4 g C m^−2^ month ^−1^), exemplified by DSL’s extreme negative residual (−17.3 g C m^−2^ month ^−1^ in July 2016) coinciding with seasonal flooding that suppressed heterotrophic respiration. Conversely, YK exhibited large positive residuals during warm growing seasons (e.g., +12.5 g C m^−2^ month ^−1^ in August 2016). Systematic biases emerged at lower altitudes, HS sustained positive residuals (>+7.0 g C m^−2^ month ^−1^ during 2019–2020), while AR showed negative deviations during drought seasons. To facilitate a clear cross-site comparison, Figure 4 synthesizes the monthly aggregated trend and residual components across the elevation gradient, whereas the daily-resolved seasonal trend decompositions for individual sites are provided in Figure S2.

**Figure 5:**
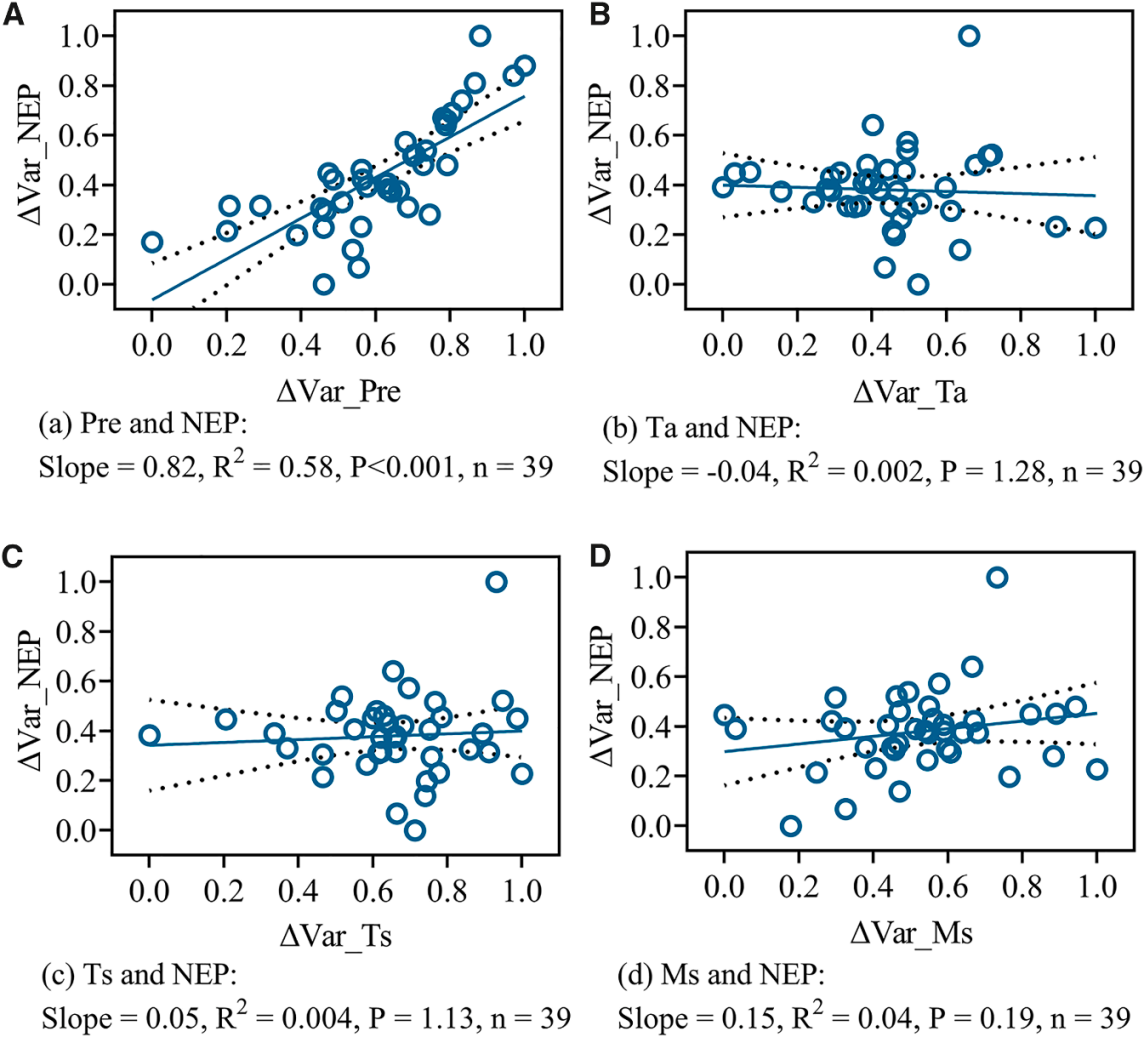
Relationship of variations in different factors and NEP Relationship of variations in precipitation (A), air temperature (B), soil temperature (C), soil moisture (D), and NEP. Δ indicates data standardization. Var represents differences between adjacent time points. Regression lines were fitted by ordinary least-squares linear regression, and *p* values are shown in the figure. *n* = 39 in each panel and represents the number of paired site-year temporal-change observations after standardization. The dotted lines represent the 95% confidence interval.

Analysis integrating environmental monitoring revealed that normalized difference vegetation index (NDVI) significantly drives NEP across ecosystems (Rst. = 0.46, *p* < 0.05), with enhanced monotonicity at higher altitudes (e.g., YK, HS, HBM, and AR). Unimodal NEP-NDVI relationships peaked in 2020 (AR) and 2017 (HBM). In swamp meadow (mean NDVI of 0.45), multi-year seasonal flooding at DSL suppressed heterotrophic respiration, bolstering carbon sequestration. Temperature critically regulated NEP, exhibiting stronger monotonicity at high-altitude sites (AR, DSL; Table S3). Ts displayed an inverse relationship to Ta (notably in HS, AR, HBM). While NEP sensitivity to temperature increased with altitude (Figure S3), its effects were modulated by terrain heterogeneity (e.g., slope aspect), vegetation types, and microbial communities (Figure S4 and Table S4). Pre significantly enhanced carbon sink capacity (*p* < 0.05; Figure 5 and Table S5), surpassing Ms in ecosystem-wide impact. Ms effects were site-specific, significant in HS and DSL swamp meadow (mean Ms of 24.7%, Figure S5) but negligible elsewhere.

**Figure 6:**
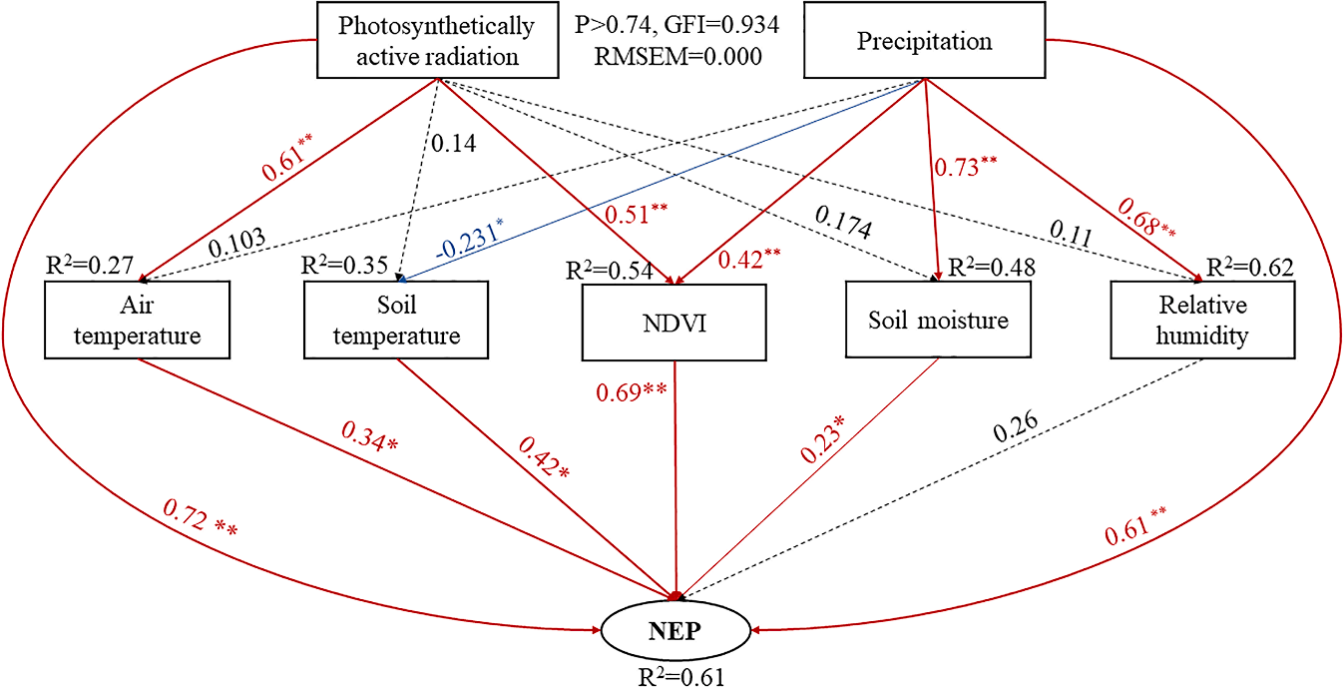
Results of the SEM The value on each arrow is the standardized path coefficient. SEM was fitted using *n* = 39 standardized site-year observations. Blue and red arrows represent significantly negative and positive correlations, respectively. Black dashed lines represent insignificant correlations. Line thickness indicates the strength of the standardized path coefficient. Asterisks denote significance of SEM path-coefficient tests: ∗*p* < 0.05 and ∗∗*p* < 0.01. CFI is the comparative fit index, RMSEM is the approximate root-mean-square error.

Structural equation modeling confirmed that hydrothermal factors (temperature, precipitation, moisture) and vegetation synergistically promoted NEP (Figures 5, 6, and S6). Improved environmental conditions amplified vegetation activity, elevating carbon sequestration. Temperature’s role as the primary limiting factor on QTP led to consistent NEP responses across ecosystems, whereas Ms effects were non-significant system-wide. NEP sensitivity to environmental change increased with altitude. All ecosystems benefited from humid conditions along the gradient (Figures 5 and S6).

By combining Spearman rank and structural equation modeling results, it was found that hydrothermal conditions (temperature, soil moisture, and precipitation) and vegetation were positively correlated with net ecosystem production. Improvements in environmental factors enhanced vegetation activity, thereby increasing net carbon sequestration. Since thermal conditions are the primary limiting factor on the QTP, the response of NEP to temperature did not vary significantly among different ecosystems (Figures 5 and S4). Research had shown that the relationship between soil moisture and NEP across different ecosystems was not significant (Table S3). In this study, NEP and soil moisture in swamp meadow and shrub ecosystems exhibited significant monotonicity. The statistical analyses consistently indicated that increased precipitation significantly enhanced carbon sink capacity (Figures 5 and 6).

Based on the limited observational data, the monotonic relationship between NEP and environmental factors indicated that, on the northeastern edge, as altitude increased, the sensitivity of NEP to environmental changes also heightened. Along the altitudinal gradient, NEP across various ecosystems uniformly benefitted from a humid environment, such as increased precipitation (Figures 6 and S4). Since temperature is the primary limiting factor in high-altitude regions, the effects of temperature fluctuations on NEP are more complex (Figure 5 and Table S4). Furthermore, the sensitivity of carbon exchange to temperature exhibited an increasing trend along the altitudinal gradient (Figure S3).

### Signal interpretation based on remote sensing data

We evaluated the remote sensing data against the eddy covariance observations at the site scale by extracting the remote sensing pixels corresponding to the eddy-covariance sites and comparing their annual flux magnitudes with tower-based estimates. It was found that the quality of remote sensing data met the requirements, with an average R^2^ of 0.73 for Re, 0.71 for gross primary production (GPP) and 0.54 for net ecosystem carbon exchange (NEE) (Figure S7). Crucially, all three empirical regression slopes were statistically significant (*p* < 0.01), with values of 0.6502 for GPP, 0.8483 for Re, and −0.4759 for NEE. Among them, the slope of Re was closest to 1, indicating that the remote sensing data of Re better matched the eddy data, followed by GPP, while the slope of NEE was farthest from 1, indicating that the NEE based on remote sensing calculations underestimated the observation results.

**Figure 7:**
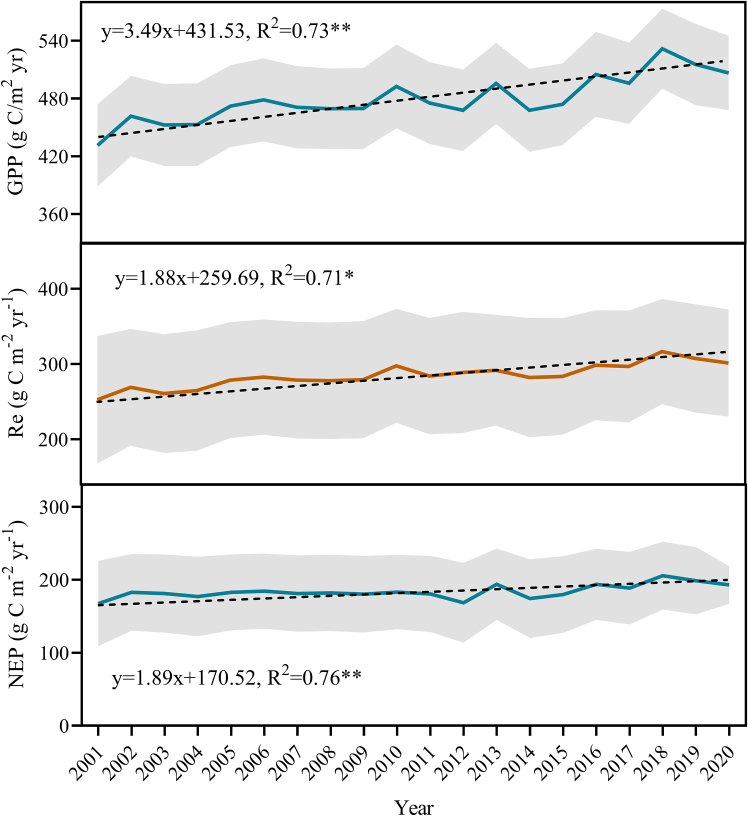
GPP, ecosystem respiration (Re), and NEP sequences based on remote sensing data in the study area during 2001–2020 The gray shaded area represents the SD range, and the dashed line represents the ordinary least-squares linear regression fit. *n* = 20 annual regional observations for each flux variable. Asterisks denote significance of the linear regression slope test: ∗*p* < 0.05 and ∗∗*p* < 0.01.

**Figure 8:**
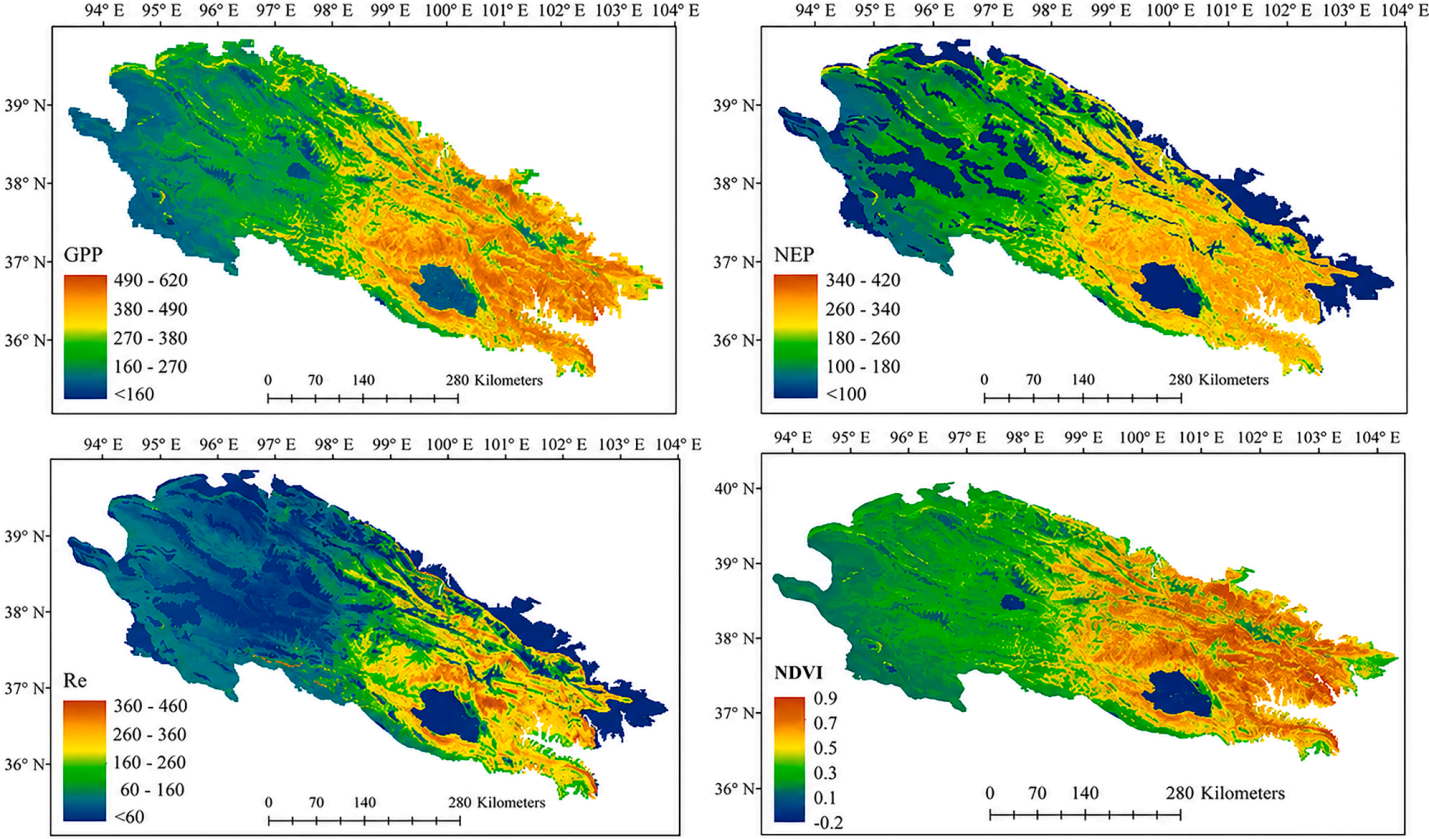
GPP, NEP, ecosystem respiration (Re) and NDVI distribution based on remote sensing data in the study area during 2001–2020

The results indicated that from 2001 to 2020, the average GPP, Re, NEP value at the northeastern edge were 469.2 ± 40.1 g C m^−2^ year^−1^, 284.8 ± 65.7 g C m^−2^ year^−1^, and 185.3 ± 44.3 g C m^−2^ year^−1^, respectively, compared with the corresponding eddy-covariance estimates of 560.1 g C m^−2^ year^−1^ for GPP, 248.9 g C m^−2^ year^−1^ for Re, and 311.2 g C m^−2^ year^−1^ for NEP. GPP, Re, and NEP all exhibited increasing trends (Figure 7), with GPP showing the fastest growth rate of 3.49 g C m^−2^ year^−2^. The difference in growth rates between NEP and Re was insignificant (*p* > 0.05). However, the growth rate of GPP significantly surpassed that of respiration, suggesting that the capacity of vegetation to absorb CO_2_ may outpace respiration in the future. This finding is consistent with the signals presented by the eddy covariance series. In addition, we also compared the temporal trends of NEP during the overlapping period (2016–2020). The OPEC data showed a growth rate of 1.49 g C m^−2^ year^−2^, corresponding to a total increase of 7.45 g C m^−2^ year^−1^ over the 5-year period. In comparison, the remote sensing data yielded a growth rate of 1.03 g C m^−2^ year^−2^, resulting in a total increase of 5.15 g C m^−2^ year^−1^. The consistently positive growth rates from both independent datasets support the reliability of the remote sensing products in capturing interannual carbon sink dynamics. However, there are still differences between them. Based on the current trend of remote sensing data, the annual NEP for the entire study area was approximately 3.72 × 10^10^ kg C year^−1^ (over an area of 20.1 × 10^4^ km^2^), although future trajectories remain uncertain due to complex environmental drivers. However, based on the results of eddy observations, the average NEP in the entire Qilian Mountains region was 6.25 × 10^10^ kg C year^−1^. The discrepancy was primarily from spatial representation. The eddy covariance sites were typically located in relatively homogeneous and highly productive alpine ecosystems, whereas the remote sensing pixels captured the full spatial heterogeneity of the entire 20.1 × 10^4^ km^2^ region, including vast areas of sparse vegetation, alpine desert, and barren lands, thereby yielding a lower but more spatially representative regional average. Spatially, the carbon sink in the study area exhibited a gradually increasing trend from northwest to southeast (including GPP and NEP), which was significantly correlated with the NDVI (Figure 8).

It should be noted that the spatially averaged NEP derived from remote sensing data differed from site based estimates. This discrepancy primarily reflected differences in spatial representativeness. Eddy sites were intentionally established in relatively homogeneous landscapes with dense vegetation cover. In contrast, remote sensing estimations integrated the entire study region and included widely distributed land cover types with low productivity, such as bare land, sparse vegetation, alpine desert, and permafrost zones. The lower regional mean NEP obtained from remote sensing was therefore expected, yet the trends presented remain consistent. The two estimation approaches were complementary. Eddy covariance data quantified the carbon sink strength at specific sites, whereas remote sensing data provided spatially explicit estimates that account for landscape scale heterogeneity.

### Comparison with other ecosystems

**Figure 9:**
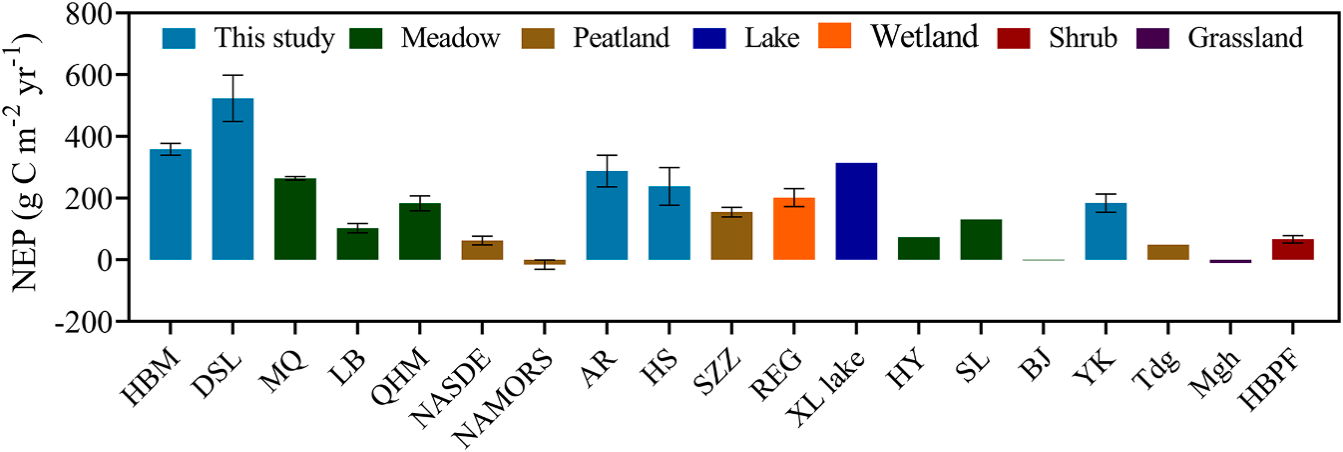
Comparison of the mean ± SD annual NEP between different ecosystems in the QTP alpine region Bars show means and error bars indicate SD. *n* represents the number of annual observations or literature-reported values used for each ecosystem comparison, exact *n* values and data sources are reported in Table S6.

To further understand the signals derived from the CO_2_ flux sequence on the northeastern edge, a comparison of eddy-covariance measurements in similar ecosystems in the QTP was conducted (Figure 9 and Table S6). The NEP of the meadow ecosystems studied (AR, YK, HBM, and DSL) was significantly higher compared to the steppe ecosystems located in the hinterland and western regions of the QTP. The sparse vegetation regions in the western part of the QTP exhibited weak carbon sink properties, with NEP ranging from −31.1 to −1.5 g C m^−2^ year^−1^. Additionally, the NEP of meadow in eastern and the hinterland of QTP was 60%–80% of the values observed in this study.

The carbon sink of different ecosystem types in the QTP ranged from 50 g C m^−2^ year^−1^ to 600 g C m^−2^ year^−1^. The carbon sink capacity of wetland and lake ecosystems in the QTP, with mean NEP of 258.3 ± 56.1 g C m^−2^ year^−1^, differed slightly from that observed at the sites included in this study.

## Discussion

This study demonstrates that the northeastern edge of the QTP acted as a significant and persistent carbon sink, as revealed by long-term eddy covariance and remote sensing data. It was important to acknowledge that the spatial scales of EC and remote sensing pixels differed substantially. The EC flux footprint (the upwind source area contributing to the measured flux) typically extended from tens to hundreds of meters depending on measurement height, wind direction, and atmospheric stability.^21^ In contrast, the GPP and Re remote sensing products used in this study had spatial resolutions of 1 km. This scale mismatch contributed to the observed discrepancy between ground-based and satellite-derived CO_2_ flux estimates, particularly in heterogeneous landscapes where the EC footprint might not fully represent the conditions averaged over a satellite pixel.^22^ Nevertheless, the consistent interannual trends observed between EC and remote sensing data (with mean R^2^ for of 0.76) suggested that the EC sites were reasonably representative of the broader landscape within their respective satellite pixels. This was consistent with the site selection criteria for the Heihe Integrated Observatory Network, which prioritized relatively flat and homogeneous fetch conditions to ensure flux measurements capture landscape-scale processes.^23^ Future studies incorporating explicit footprint modeling and footprint-weighted satellite retrievals would allow a more rigorous quantification of spatial representativeness.^22^

Our results showed a pronounced altitudinal gradient in the increase in NEP, with the most substantial enhancements occurring at high-altitude sites (Figure 4). This finding suggested that, contrary to some expectations, high-altitude ecosystems currently contribute disproportionately to the strengthening of the regional carbon sink. The short-term variability in NEP, indicated by the residual components, was also greatest at high altitudes (Figures 4 and S2), highlighting heightened sensitivity to environmental fluctuations. Based on observational data, we hypothesized that future warming and increased humidification may further enhance the carbon sink capacity of these high-altitude regions,^15^ whereas drought events may weaken this capacity.^24^

The positive monotonic relationship between precipitation and NEP identified across our sites (Tables S1 and S3) confirms that humidification is beneficial for carbon sequestration in this region, consistent with findings from other cold ecosystems.^25^^,^^26^ The extreme positive NEP residual at DSL during flooding (Figure 4) provides a clear example of how abundant water can enhance carbon uptake. We propose that the mechanism behind this involves two processes, as supported by our data and literature. First, increased precipitation and soil moisture can inhibit the metabolic activity of aerobic organisms,^27^ thereby reducing heterotrophic respiration. This is consistent with our structural equation model (SEM) results, which showed a limited direct role of Ms system-wide, but significant effects at the swamp meadow sites (HS and DSL) where water saturation is common. This phenomenon was also observed in peatlands, Arctic tundra ecosystems, and Plateau lakes.^28^^,^^29^^,^^30^ Second, adequate precipitation supports plant growth in high-altitude areas, enhancing GPP, as reflected in the strong coupling between NDVI and NEP in our study. This may help mitigate carbon losses from soils under warming.^31^

The observed altitudinal pattern in NEP trends, whereby carbon sink strengthening was most pronounced at high elevations, can be interpreted through the lens of growing season extension and substrate dynamics. The extended growing season resulting from climate change was a widespread phenomenon,^32^ which likely prolonged vegetation activity and indirectly enhances carbon uptake,^33^ an effect that might be particularly beneficial at high altitude. Our data further suggested that compared to low-altitude regions, high-altitude areas were constrained by limited nutrients and environmental conditions necessary for vegetation growth, inhibiting carbon and substrate consumption.^15^^,^^27^ This limitation favored the sequestration of photosynthetically fixed carbon. Furthermore, the soil above 4100 m was predominantly cold desert soil with extremely low organic carbon storage.^34^ Consequently, microbial decomposition was inherently limited by substrate availability. However, the large residual fluctuations we observed at high-altitude sites (YK, DSL) also indicated that these ecosystems were more susceptible to environmental change. This susceptibility, coupled with the fact that vegetation cover on the QTP was increasing,^35^^,^^36^ which enhanced net carbon uptake, providing a basis for heterotrophic respiration, introducing uncertainty regarding the potential reversal of the current carbon sink trend under warming.

This interplay between carbon uptake and emission was also reflected in the variations among underlying surface types. In our study, the shrub ecosystem (HS) exhibited the highest rates of carbon exchange. This pattern could be elucidated by the ecological strategies of shrubs, which typically exhibited rapid growth rates but short growth cycles,^37^ which restricted the duration of carbon storage through the swift accumulation of biomass during the growing season, providing a readily available substrate for microbial decomposition. Additionally, shrubs and meadows exhibited distinct resource allocation patterns. The proportion of aboveground biomass in shrubs was relatively high,^37^ which might indirectly influence the transfer of carbon to the soil and enhance the decomposition of organic matter. In contrast, our results showed that meadows had higher net carbon absorption rates compared to shrubs, although with lower emission rates, which could be attributed to extended growth cycle that facilitated more sustained carbon sequestration. Nevertheless, drought stress directly impacted plant stomatal conductance, reducing carbohydrate conversion rates,^38^ and consequently weakening carbon sequestration^26^^,^^39^ while potentially increasing microbial heterotrophic respiration. The negative deviations we observed at site AR during drought seasons align with this understanding. Therefore, in the context of warming, carbon emissions from arid areas, sparsely vegetated regions, and specialized underlying surfaces would increase, potentially turning these areas into carbon sources due to the absence of robust carbon sequestration mechanisms.

The characteristics and trends of NEP on the northeastern edge of the QTP were analyzed using continuous OPEC observation data. The results indicated that the region continued to act as a moderate and significant net carbon sink, with mean NEP of 311.2 ± 127.9 g C m^−2^ year^−1^ (mean ± SD of site-level annual values during same period), and average NEP calculated based on remote sensing data was 185.3 ± 44.3 g C m^−2^ year^−1^. This quantification underscores the important role of this alpine ecosystem in the regional carbon budget. As a typical area of permafrost degradation, climate change exacerbated carbon loss. In the context of warming and increased humidity, carbon emissions in high-altitude areas may be offset by seasonal and interannual carbon uptake, thereby maintaining a carbon surplus.

This study provided a comprehensive assessment of the carbon sequestration capacity across the entire Qilian Mountains region (covering a total area of 20.1 × 10^4^ km^2^ like Figure 1). Both EC observations and remote sensing estimations demonstrated that the region acted as a carbon sink, corresponding to a total regional NEP of 6.25 × 10^10^ ± 2.57 × 10^10^ kg C year^−1^ (for EC) and 3.72 × 10^10^ ± 1.3 × 10^10^ kg C year^−1^ (for remote sensing). The discrepancy between the two methods was reasonable and primarily stems from spatial representation. EC footprints typically capture homogeneous, highly productive hotspots, whereas remote sensing pixels integrate the full spatial heterogeneity of the entire region, thereby yielding a lower but more representative regional average carbon sink. These results greatly contribute to achieving carbon neutrality and reduce the reliance on more costly engineered carbon removal strategies, while simultaneously lowering the long-term socio-economic costs associated with ecosystem degradation in this region.

### Limitations of the study

This study provides key insights into the carbon dynamics of the Qilian mountains, yet some limitations should be considered, which also point to valuable directions for future research.

The findings were primarily representative of meadow and shrub ecosystems. To achieve a comprehensive regional carbon budget, future efforts should incorporate a wider variety of ecosystem types prevalent in the Qilian mountains, including forests (accounting for 10.1% of the total area, e.g., *Juniperus przewalskii*, *Picea crassifolia*, which dominate north-facing slopes at mid-elevations), as well as alpine deserts (accounting for 22.5% of the total area). In particular, forests represent an important but currently under-sampled component of the regional carbon cycle, and their inclusion would significantly improve the representativeness of regional carbon sink estimates.

Understanding of the underlying biogeochemical mechanisms was constrained by the available data. The discussion on microbial communities and soil carbon pools remained inferential, as these variables were not directly measured. Future interdisciplinary research that integrates isotopic techniques, microbial genomics, and soil carbon stock measurements with continuous flux observations is essential to unravel the “black box” of belowground processes.

While we identified trends over the past decade, the long-term series of carbon sink remained uncertain. Extending these time series and integrating them with process-based ecological models is critical for forecasting the carbon sink capacity of the alpine region under future climate scenarios.

## Resource availability

### Lead contact

Requests for further information and resources should be directed to and will be fulfilled by the lead contact, Yiwen Liu (liuyiwen@nieer.ac.cn).

### Materials availability

This study did not generate new materials.

### Data and code availability

• EC data and environmental factors data used in this study can be applied for download at the National Tibetan Plateau/Third Pole Environment Data Center (http://data.tpdc.ac.cn, Data: Dataset of Heihe integrated observatory network, doi: https://doi.org/10.11888/Terre.tpdc.302895) and National Ecosystem Science Data Center, National Science & Technology Infrastructure of China (http://www.nesdc.org.cn, doi:https://doi.org/10.57760/sciencedb.06764, and https://doi.org/10.57760/sciencedb.06763). Remote sensing data of gross primary productivity and ecosystem respiration were obtained from the LAADS (the National Tibetan Plateau/Third Pole Environment Data Center, doi:https://doi.org/10.11888/Terre.tpdc.300229) and the National Tibetan Plateau/Third Pole Environment Data Center (http://data.tpdc.ac.cn). NDVI data was from the MODIS product (https://ladsweb.modaps.eosdis.nasa.gov).
• This study does not report original code.
• Any additional information required to reanalyze the data reported in this study is available from the lead contact upon request.

## Acknowledgments

This study was funded by Youth Cross-Disciplinary Innovation Team of State Key Laboratory of Ecological Safety and Sustainable Development in Arid Lands (no. 2024000689), the Central Government for Local Science and Technology Development Project (no. 25ZYJA019), the Gansu Provincial Science and Technology Planning Project (no. 24ZD13FA004), the Provincial Natural Science Foundation of Hunan (2025JJ60225), and 10.13039/501100013494West Light Foundation, Chinese Academy of Sciences (Study on the Warming Effects of Typical Grasslands Based on Habitat Control Devices).

## Author contributions

C.H. and Y.L.: writing – original draft and formal analysis; X.W. and G.L.: resources and data curation; Z.L.: methodology; R.C.: writing – review and editing and conceptualization.

## Declaration of interests

The authors declare no competing interests.

## STAR★Methods

### Key resources table

**Table:** 

REAGENT or RESOURCE	SOURCE	IDENTIFIER
**Deposited data**		

Raw and analyzed data	the National Tibetan Plateau / Third Pole Environment Data Center	https://doi.org/10.11888/Atmos.tpdc.300632 https://doi.org/10.11888/Atmos.tpdc.300654 https://doi.org/10.11888/Atmos.tpdc.300625 https://doi.org/10.11888/Atmos.tpdc.300626 https://doi.org/10.11888/Atmos.tpdc.300652 https://doi.org/10.11888/Atmos.tpdc.300628
Raw and analyzed data	National Science & Technology Infrastructure of China	https://doi.org/10.57760/sciencedb.06764 https://doi.org/10.57760/sciencedb.06763
Raw and analyzed data	MODIS	https://ladsweb.modaps.eosdis.nasa.gov

**Software and algorithms**		

Python 3.12	Open-source software	https://www.python.org/

### Method details

#### Study area

The QLM is located in the mid-latitude northern temperate zone (94°10'-103°04' E to 35°50'-39°19' N, with an altitude range from 2100 to 5800 m), at the convergence of the QTP and the Loess Plateau, serving as a crucial ecological barrier on the northeastern edge of the QTP (Figure 1). Due to the altitude and terrain of the QLM, a significant vertical landscape gradient is observed within the study area. Furthermore, the climate and vegetation types exhibit considerable diversity, ranging from arid climates with sparse vegetation in dry areas to humid climates with forests in mountainous regions, establishing it as a quintessential vegetation-desert ecotone.

#### Eddy covariance data

To understand the signals represented by CO_2_ flux trends at the northeastern edge of the QTP, open-path eddy covariance data from five distinct ecosystems were utilized (Figure 1, Tables 1 and S1 in Text S1), sourced from the National Tibetan Plateau / Third Pole Environment Data Center (https://data.tpdc.ac.cn) and the National Ecosystem Science Data Center, National Science & Technology Infrastructure of China (http://www.nesdc.org.cn). The ecosystem types include meadow (AR, HBM, and YK), swamp meadow (DSL), and shrub (HS). This region is also a typical area of degraded permafrost,^40^ characterized by ample soil moisture and nutrients.

The OPEC system comprises a three-dimensional anemometer (CSAT3, Campbell Scientific, UT, USA), an open-path infrared gas analyzer (Li-7500, Li-COR Inc, Lincoln, NE, USA), and a data logger. The original collection frequency for gas density was 10 Hz, and the data were processed into 30-minute flux averages using standardized programs. The post-processed data underwent a rigorous preprocessing pipeline, which included the removal of outliers, correction for time lags, application of a double coordinate rotation, and compensation for frequency response. Additionally, corrections for sonic virtual temperature and density fluctuations were applied.^41^ Each flux value was subjected to a quality assessment based primarily on tests for atmospheric stationarity (Δst) and the integral turbulence characteristics (ITC). For detailed information about these preprocessing procedures, please refer to relevant publications.^23^^,^^33^^,^^42^

To ensure the reliability of the OPEC measurements underpinning CO_2_ flux estimates, we assessed the energy balance closure status of the study sites. While recent dataset publications have excluded soil heat flux observations due to uncertainties in permafrost region,^42^ prior analyses of the same observation network provide robust validation. Specifically, Liu et al.^43^ evaluated the energy balance at the AR. They reported an energy balance closure regression slope of 0.86 (R^2^ = 0.89) in 2008 and 0.73 (R^2^ = 0.88) in 2009, with corresponding cumulative energy balance ratios of 0.89 and 0.85. These closure rates are consistent with the 0.70–0.90 range widely observed across global FLUXNET sites^44^ and confirm that the turbulent flux data from this network are of sufficient quality to support the calculation of annual carbon budgets. In addition, the difference between the average sensible and latent heat flows (regression slope) at was 3% and 10% for the study area. The difference in sensible flow was 2%, and the difference in net radiation was less than 1%. The measurement results of EC and thermal flow were consistent with a regression slope of about 3%, demonstrating the reliability of the measurement data.^33^

#### Data processing and gap filling

Net ecosystem carbon exchange (NEE) was partitioned into gross primary production (GPP) and ecosystem respiration (Re), with net ecosystem productivity (NEP) defined as NEP = –NEE. Nighttime Re was estimated using the exponential relationship between nighttime NEE (when NEE = Re) and soil temperature (Equation 1). To account for the pronounced seasonal differences in ecosystem physiology across the study region, the parameters a and b were derived separately for the growing season and the non-growing season. Daytime Re was estimated from the intercept parameter γ of the rectangular hyperbola light-response curve (Equation 2). Following the approach of Lasslop et al.^45^ and as described in Aubinet et al.,^46^ the light-response curve was fitted to daytime NEE and downward shortwave radiation, which was used as a proxy for photosynthetically active radiation (PPFD). The use of shortwave radiation in place of PPFD is a standard practice in eddy covariance studies when direct PPFD measurements are unavailable. To obtain a robust estimate of γ, which represents daytime Re at zero light, the light-response curve (Equation 2) was fitted to daytime NEE data, where daytime periods were defined by downward shortwave radiation exceeding 20 W m^-2^ pooled within a 7-day moving window. Separate model parameters (α, β and γ) were estimated for the growing season and non-growing season to capture seasonal variations in photosynthetic capacity and daytime respiration rates.

(Equation 1) $$Re\_nighttime=ae^{bT_{s}\_nighttime}$$

(Equation 2) $$NEE\_daytime=-\frac{\alpha \times \beta \times PPFD}{\alpha \times PPFD+\beta }+\gamma$$

where *Re_nighttime* is the Re during nighttime, *T*_*s_nighttime*_ is the soil temperature during nighttime, *a* and *b* are the fitting parameters, *NEE_daytime* is the net ecosystem carbon exchange during daytime, *α* and *β* are the fitting parameters, which represent the ecosystem radiation-use efficiency and the maximum net CO_2_ uptake rate of the ecosystem at light saturation, respectively. *γ* is the intercept parameter at zero light, which characterizes ecosystem respiration, *PPFD* is the photosynthetic photon flux density, replacing by the downward shortwave radiation in the study.

To estimate Re for all daytime periods, the relationship between the derived γ values and soil temperature was established using an exponential function (Equation 1), again stratified by growing and non-growing seasons. This temperature-dependent model was then applied to estimate Re for all daytime half-hourly intervals. GPP was subsequently calculated as the difference between Re and NEE (GPP = Re – NEE). The daily, monthly, and annual integrated flux data were then computed for further analysis.

#### Remote sensing data

To further capture the signals associated with CO_2_ flux, we validated the remote sensing data with observational sequences from OPEC in this study using 8-day cumulative GPP data from MOD17A2H and MYD17A2H (https://ladsweb.modaps.eosdis.nasa.gov/missions-and-measurements/products/MYD17A2H/, https://ladsweb.modaps.eosdis.nasa.gov/missions-and-measurements/products/MOD17A2H). The 8-day cumulative GPP data were obtained from the Terra MOD17A2H and Aqua MYD17A2H products. Both products are Level-4 gridded datasets and have been validated to Stage-3, indicating that their accuracy has been assessed and uncertainties well-established via independent global measurements. The products are based on the MODIS Photosynthesis algorithm (Equation 3).

(Equation 3) $${GPP=\varepsilon }_{\max}\times f\left(T_{\min}\right)\times f\left(VPD\right)\times APAR$$

where ε_max_ is the maximum light use efficiency, *f*(*T*_*min*_) and *f*(*VPD*) are attenuation scalars representing limitations imposed by daily minimum air temperature and daytime mean vapor pressure deficit, respectively, and *APAR* is the absorbed photosynthetically active radiation. The 8-day cumulative GPP data are generated by summing the daily estimates over each 8-day compositing period. To ensure data quality, pixels affected by cloud contamination were excluded using the quality assessment flags provided with the products.

Additionally, Re data based on ReRSM (https://data.tpdc.ac.cn/), which form a cross-validation with OPEC data. The dataset provides monthly gridded Re estimates for the Tibetan Plateau at 0.05° spatial resolution, which is generated using Random Forest machine learning model trained on flux tower Re observations from eddy covariance sites across the Tibetan Plateau. The model integrates multiple environmental predictors, including soil temperature, soil moisture, normalized difference vegetation index (NDVI), and land cover type, to upscale site-level Re measurements to regional scales (https://doi.org/10.11888/Terre.tpdc.300229).

The environmental factors monitored synchronously included air temperature (Ta), soil temperature (Ts), precipitation (Pre), soil moisture (Ms), photosynthetically active radiation (PAR), and relative humidity (RH). Environmental factors data were obtained from the the National Tibetan Plateau / Third Pole Environment Data Center (http://data.tpdc.ac.cn).^23^^,^^47^ Additionally, the MODIS 16-day composite NDVI product (MOD13A2) was utilized (https://ladsweb.modaps.eosdis.nasa.gov) to investigate the influence of vegetation characteristics on carbon sinks. We conducted spatial resampling on all remote sensing data to achieve a spatial resolution of 1 km × 1 km.

#### Statistical analysis

To quantify the long-term trends and short-term anomalies in NEE, Seasonal-Trend decomposition using Loess (STL) was applied to monthly NEE time series from all sites (AR, DSL, HBM, HS, YK) during series period (Spring: March, April and May, Summer: June, July and August, Autumn: September, October and November, Winter: December, January and February). STL decomposes a time series *Y*_*t*_ into three additive components (Equation 4). The trend and seasonal components were iteratively estimated via Locally Weighted Scatterplot Smoothing (LOESS). For each observation *t*, LOESS computes a weighted polynomial regression (Equations 5 and 6). The complex monthly NEE time series was decomposed into its additive components of trend, seasonality, and residuals by applying the STL method. Structural equation modeling (SEM) and Spearman rank tests were also employed to explore the relationships between NEP and various environmental factors.

(Equation 4) $$Y_{t}=T_{t}+S_{t}+R_{t}$$

(Equation 5) $$\bar{T_{t}}=\sum_{i=1}^{n}w_{i}\left(t\right)\times y_{i}$$

(Equation 6) $$w_{i}\left(t\right)={\left(1-{\left|\frac{t-i}{d\left(t\right)}\right|}^{3}\right)}^{3}for\;\;|t-i|<d\left(t\right)$$

where *T*_*t*_ is trend component, *S*_*t*_ is seasonal component (periodic fluctuations, fixed at 12 months for annual cycle), *R*_*t*_ is residual component, *W*_*i*_*(t)* is weights, *d(t)* is distance to the α-th nearest neighbor (α=1.5×cycle length). Robust iterations (3 outer, 1 inner) minimized outlier influence. All analyses used the statsmodels STL implementation in Python v3.12.

The Spearman rank test is a nonparametric statistical method used to measure rank monotonicity between variables, denoted as Rst. in this study. This method involves converting the original observations of the two variables into ranks, ordered from smallest to largest and assigned a ranking. It is important to emphasize that, to eliminate multicollinearity in the statistical analysis, the original data were processed using regularization standardization (LASSO). Detailed information was outlined in Text S2 (Table S2) of the supplemental information.

### Quantification and statistical analysis

All statistical analyses and data visualizations were performed using Python 3.12.

Temporal trends in Figures 2, 3, and 7 were evaluated by ordinary least-squares linear regression. For Figures 2 and 3, n represents the number of annual or annual-seasonal site-level NEP observations used in each regression (AR, *n* = 7; DSL, *n* = 7; HBM, *n* = 6; HS, *n* = 10; YK, *n* = 7). For Figure 7, n represents the 20 annual regional remote-sensing observations from 2001 to 2020 for each flux variable (GPP, Re, and NEP). For Figure 4, n represents the monthly aggregate observations used in STL decomposition (AR, *n* = 84; DSL, *n* = 84; HBM, *n* = 72; HS, *n* = 120; YK, *n* = 84).

Associations between standardized temporal changes in NEP and environmental variables in Figure 5 were assessed by ordinary least-squares linear regression using *n* = 39 paired site-year temporal-change observations, n is shown in each panel and represents the number of paired observations after standardization. Spearman rank tests were used to evaluate monotonic associations between NEP and environmental drivers, and Rst. denotes the Spearman rank correlation coefficient. Site-level Spearman coefficients and *p* values are reported in Table S3.

Structural equation modeling (SEM) was used to test direct and indirect paths among hydrothermal variables, NDVI, and NEP in Figure 6 using *n* = 39 standardized site-year observations. The statistical tests were two-sided, *p* < 0.05 was considered statistically significant. Values reported in the text and figure legends are mean ± SD unless otherwise noted, regression lines are shown with 95% confidence intervals where indicated. Asterisks indicate statistical significance as follows, ∗*p* < 0.05 and ∗∗*p* < 0.01. Statistical details, including test names, exact *n* values and what n represents, definitions of center and dispersion, and *p*-value thresholds, are provided in this section, the Results, the figure panels, figure legends, and Tables S3–S6.
