# Supplementary material for: Signal of carbon exchange in Qilian mountain based on eddy covariance and remote sensing data
Source: iScience. 2026 Jul 27;29(8):116949. doi: 10.1016/j.isci.2026.116949 (PMC13446361; doi:10.1016/j.isci.2026.116949)
Supplement: Supplementary file 1 [file mmc1.pdf]

iScience, Volume *29*

Supplemental information

Signal of carbon exchange in Qilian mountain based on eddy covariance and remote sensing data

Chuntan Han, Yiwen Liu, Xiaobo Wang, Zhangwen Liu, Guohua Liu, and Rensheng Chen

Contents of this file

Text S1 and S2

Figures S1 to S7

Table S1 and S6

Text S1 (Eddy covariance data details, Related to Figure 1 and Table 1)

The open-path eddy covariance data was from the National Tibetan Plateau / Third Pole Environment Data Center (https://data.tpdc.ac.cn, hereinafter referred to as NTP) and National Ecosystem Science Data Center, National Science & Technology Infrastructure of China (http://www.nesdc.org.cn, hereinafter referred to as NES). Specifically, eddy data at YK, AR, and DSL come from the NTP, while data at HBM and HS come from the NES. Due to the fact that the eddy data series provided by NES at HS stie does not include observations from 2014, 2015, and 2019, we combined the long sequence data based on surface observations provided by NTP. The raw data clearly attributable to instrument malfunction, such as measurements of −9999 or -6999, negative values of net ecosystem carbon exchange during the nighttime, and data associated with a friction velocity (u*) below 0.1 m/s was discarded.[1,2]

Data Filling: First, the missing time series of eddy data were screened to determine the continuity. Data that were less than 2 hours apart were reconstructed using linear interpolation. For data that were more than 2 hours apart, data for the growing season and non-growing season were reconstructed during the daytime and nighttime. The methods used were soil temperature index method (nighttime, Eq. 1) and rectangular hyperbola light-response curve (daytime, Eq. 2).

Table S1 Data identification of each site

Site	OPEC data DOI	Environmental data DOI
AR	https://doi.org/10.11888/Atmos.tpdc.300632	https://doi.org/10.11888/Atmos.tpdc.300626
YK	https://doi.org/10.11888/Atmos.tpdc.300654	https://doi.org/10.11888/Atmos.tpdc.300652
DSL	https://doi.org/10.11888/Atmos.tpdc.300625	https://doi.org/10.11888/Atmos.tpdc.300628
HBM	https://doi.org/10.57760/sciencedb.06764	
HS	https://doi.org/10.57760/sciencedb.06763	

Table S2 Quality information of eddy data at each site

Site	Ecosystem	Lon.	Lat.	Alt. (m)	Time range	Data coverage (%)	High coverage (%)	Filled coverage (%)
AR	Meadow	100.46	38.04	3033	2016-2022	97	84	13
HBM	Meadow	101.38	37.75	3250	2015-2020	100	90	10
YK	Meadow	100.24	38.01	4148	2016-2022	98	89	9
DSL	Swamp meadow	98.94	38.84	3739	2016-2022	97	86	11
HS	Shrub	101.21	37.48	3180	2011-2020	95	92	3

Note: Lon. means longitude. Lat. means latitude. Alt. means altitude. Data coverage means percentage of the period with available data. High coverage means the percentage of high-quality data. Filled coverage means

The percentage of gap-filled data. In some stations, the coverage of data is not 100%, which is due to the existence of Eddy Data and environmental factor data are missing, which cannot be filled. Mainly reflected in the instrument damage or power supply system problems.

Text S2 (Detailed information on Spearman rank test, Related to *STAR★METHODS, Statistical analysis* in Manuscript)

The Spearman rank coefficient ranges from -1 to 1, where -1 indicates complete negative correlation, 1 indicates complete positive monotonicity correlation, and 0 indicates no monotonicity correlation. Test whether there is a monotonic relationship between two variables. Spearman rank coefficient can be used to determine whether there is a monotonic relationship between different variables, that is, whether the increase of one variable is accompanied by the increase or decrease of another variable (monotonic relationship differs from simple linear correlation). This is useful for observing trends or patterns between data. This method can replace the Pearson correlation coefficient and facilitate the observation of more subtle changes.

$$Rst.=\frac{\frac{1}{n}\sum_{i=1}^{n}\left(R(x_i)-\overline{R(x)}\cdot R(y_i)-\overline{R(y)}\right)}{\sqrt{\left(\frac{1}{n}\sum_{i=1}^{n}\left(R(x_i)-\overline{R(x)}\right)^2\right)\cdot\left(\frac{1}{n}\sum_{i=1}^{n}\left(R(y_i)-\overline{R(y)}\right)^2\right)}}$$

Where $R(x)$ and $R(y)$ represent the rank of x and y, $\overline{R(x)}$ and $\overline{R(y)}$ represent the average rank.

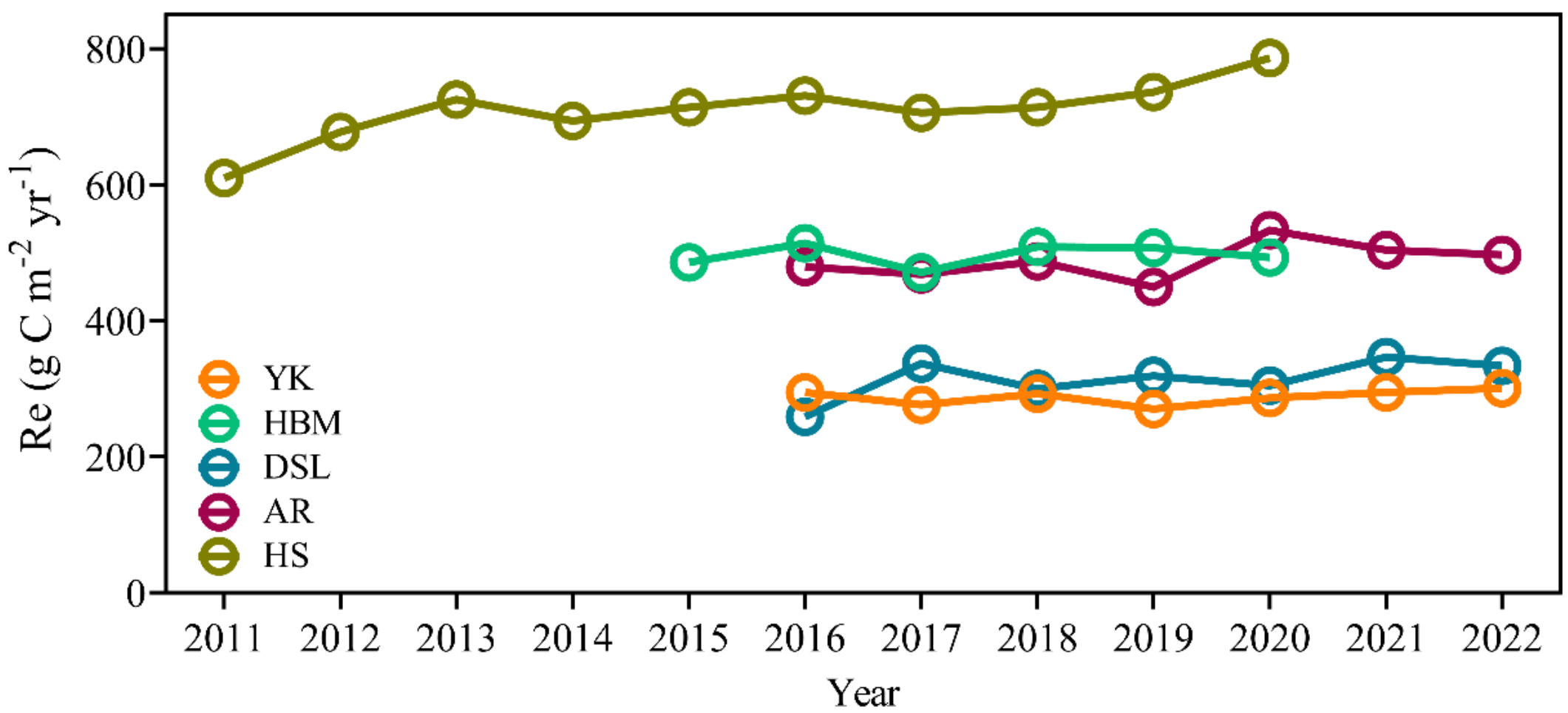

Figure S1 Observational trends of ecosystem respiration in the northeast of the Qinghai-Tibet Plateau at the annual scale. **Related to Figure 2.**

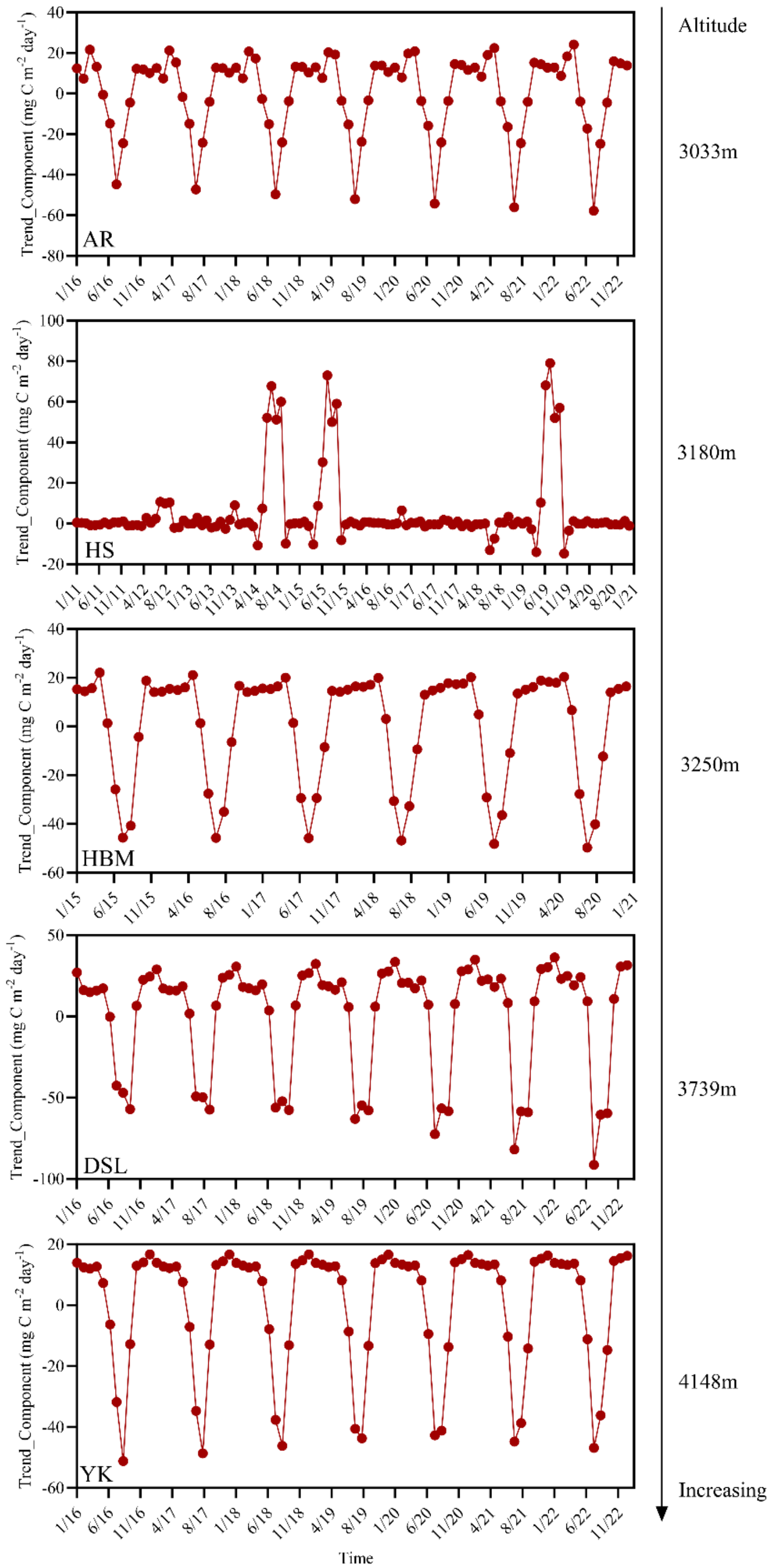

Figure S2 Continuous seasonal-trend decomposition of net ecosystem productivity time series at daily scale for the five study sites (YK, DSL, HBM, HS, and AR). For each site, the panels plot the decomposed components including the net ecosystem productivity component values after each site had undergone trend decomposition. **Related to Figure 4.**

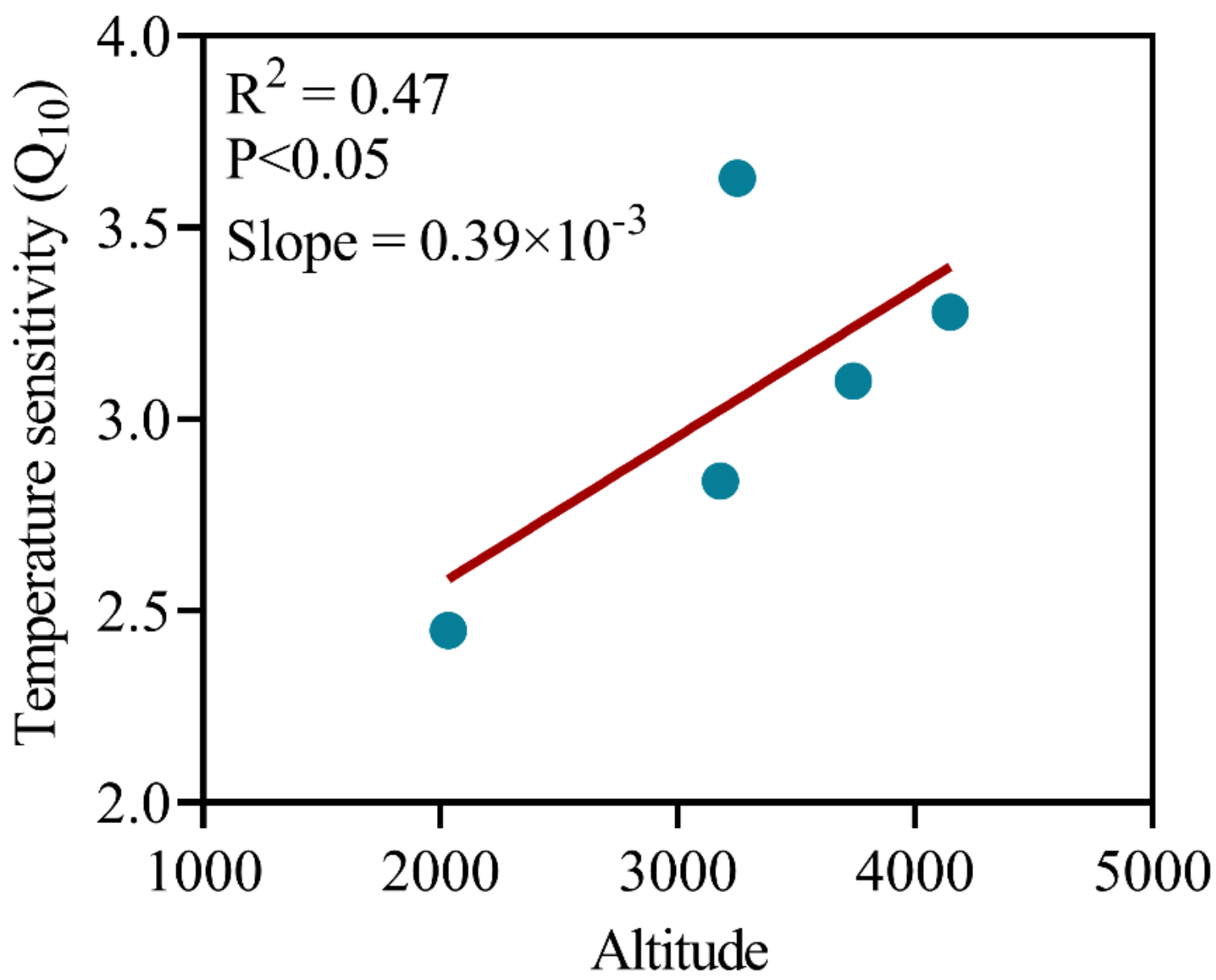

Figure S3 The relationship between temperature sensitivity (Q_{10}) of carbon exchange in ecosystem and altitude. **Related to Figure 4.**

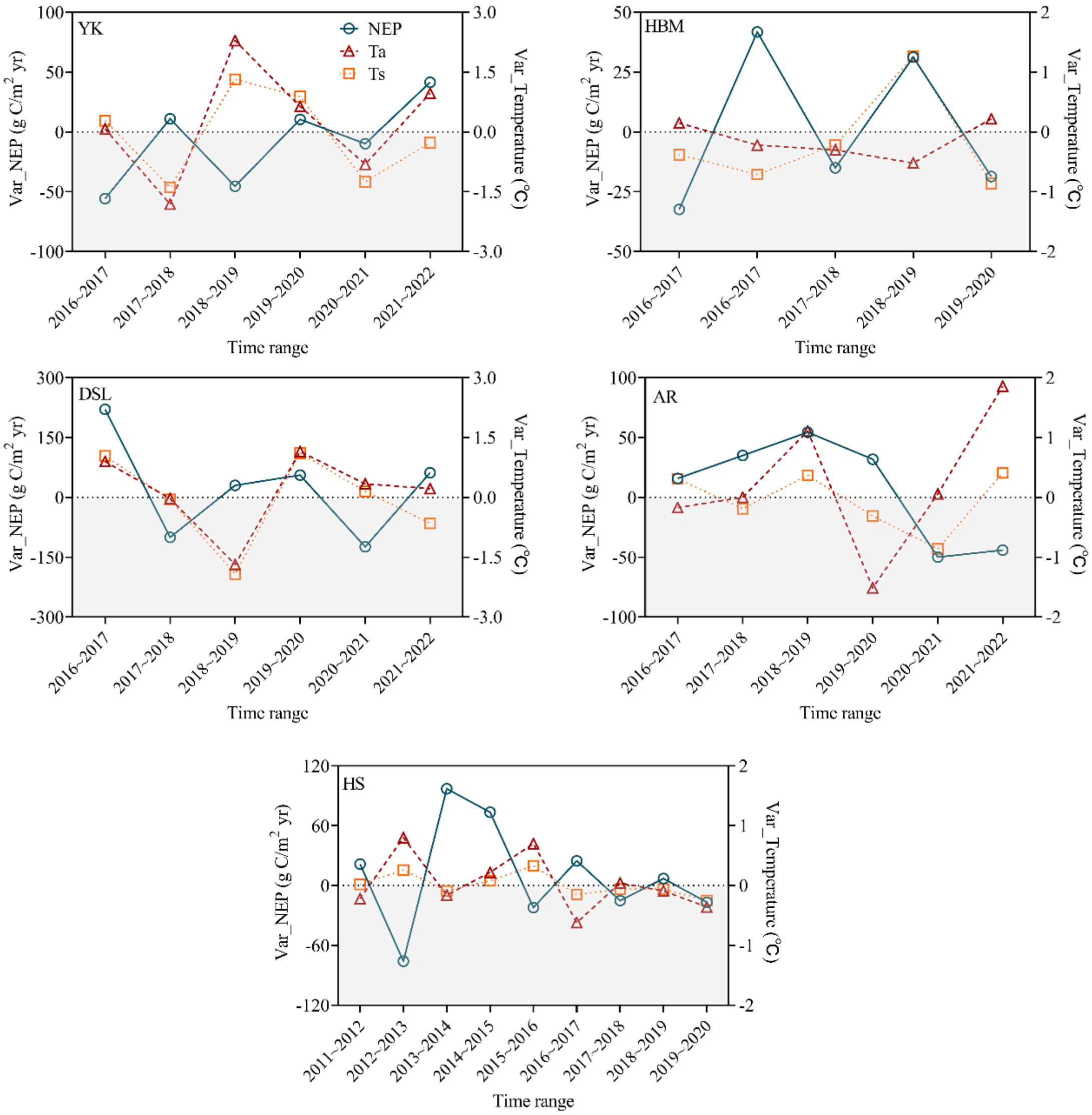

Figure S4 Comparison of variations in temperature and NEP during the observation period. Temperature includes air temperature (Ta) and soil temperature (Ts). Areas less than 0 and greater than 0 in the figure are represented by gray and white respectively. When the variable difference is distributed in the white area, indicating that the variable has increased compared with the previous year. When the variable difference is distributed in the gray area, indicating that it decreases. **Related to Figure 5.**

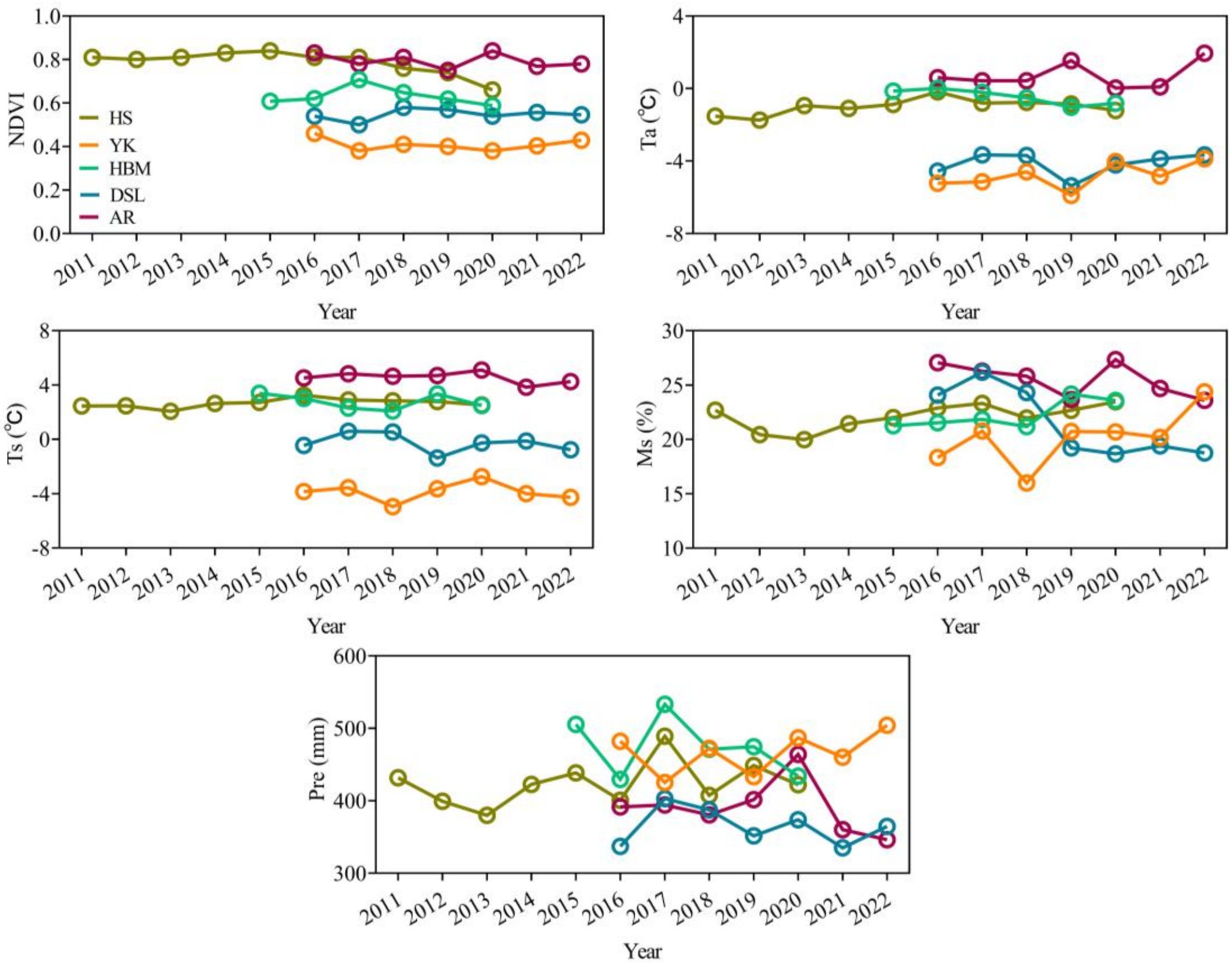

Figure S5 Trends in environmental factors of different ecosystems during observation period. Ta is air temperature, Ts is soil temperature, Ms is soil moisture and Pre is precipitation. **Related to Figure 6.**

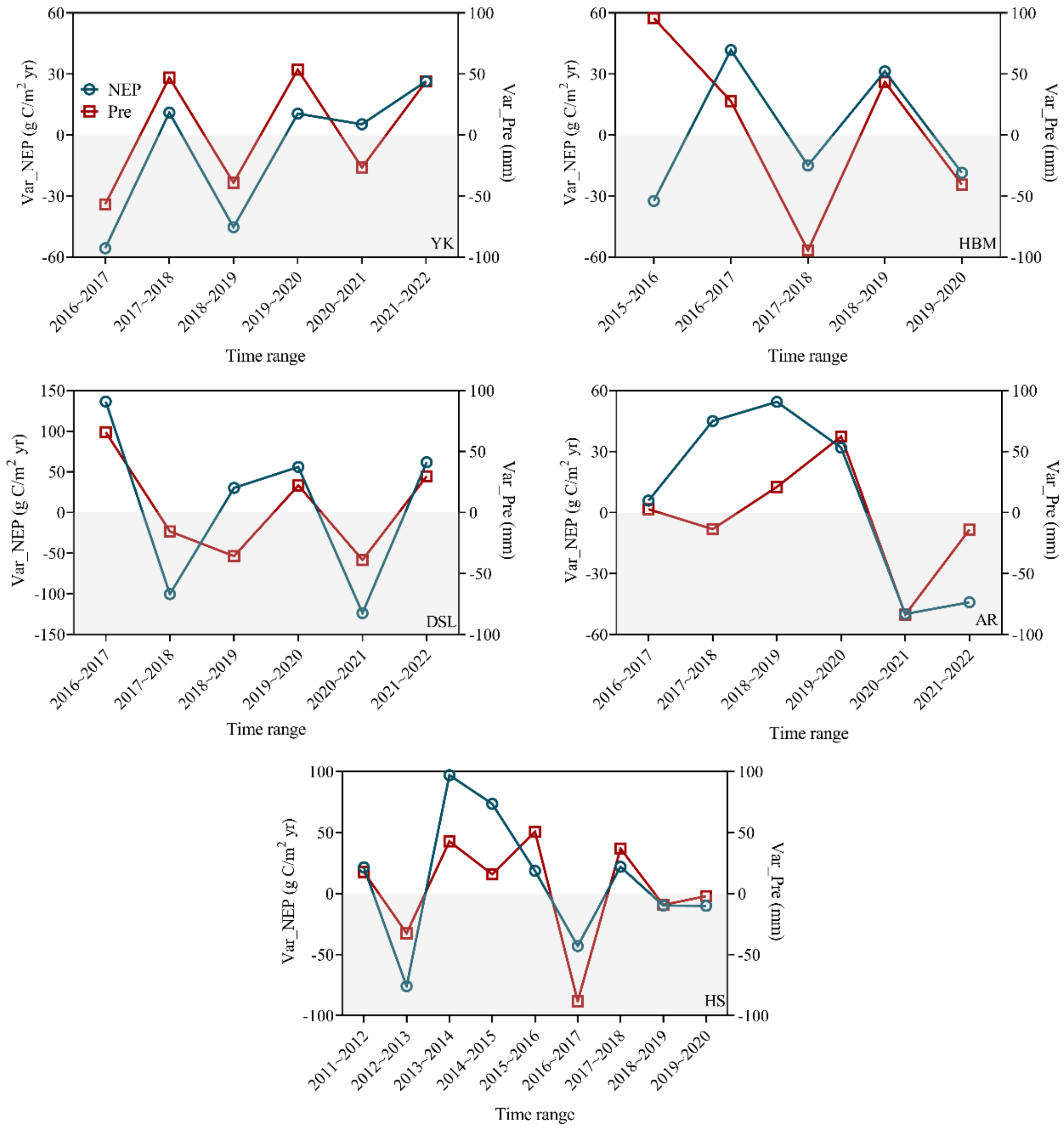

Figure S6 Comparison of variations in precipitation and NEP during the observation period. Areas less than 0 and greater than 0 in the figure are represented by gray and white respectively. When the variable difference is distributed in the white area, indicating that the variable has increased compared with the previous year. When the variable difference is distributed in the gray area, indicating that it decreases. **Related to Figure 5.**

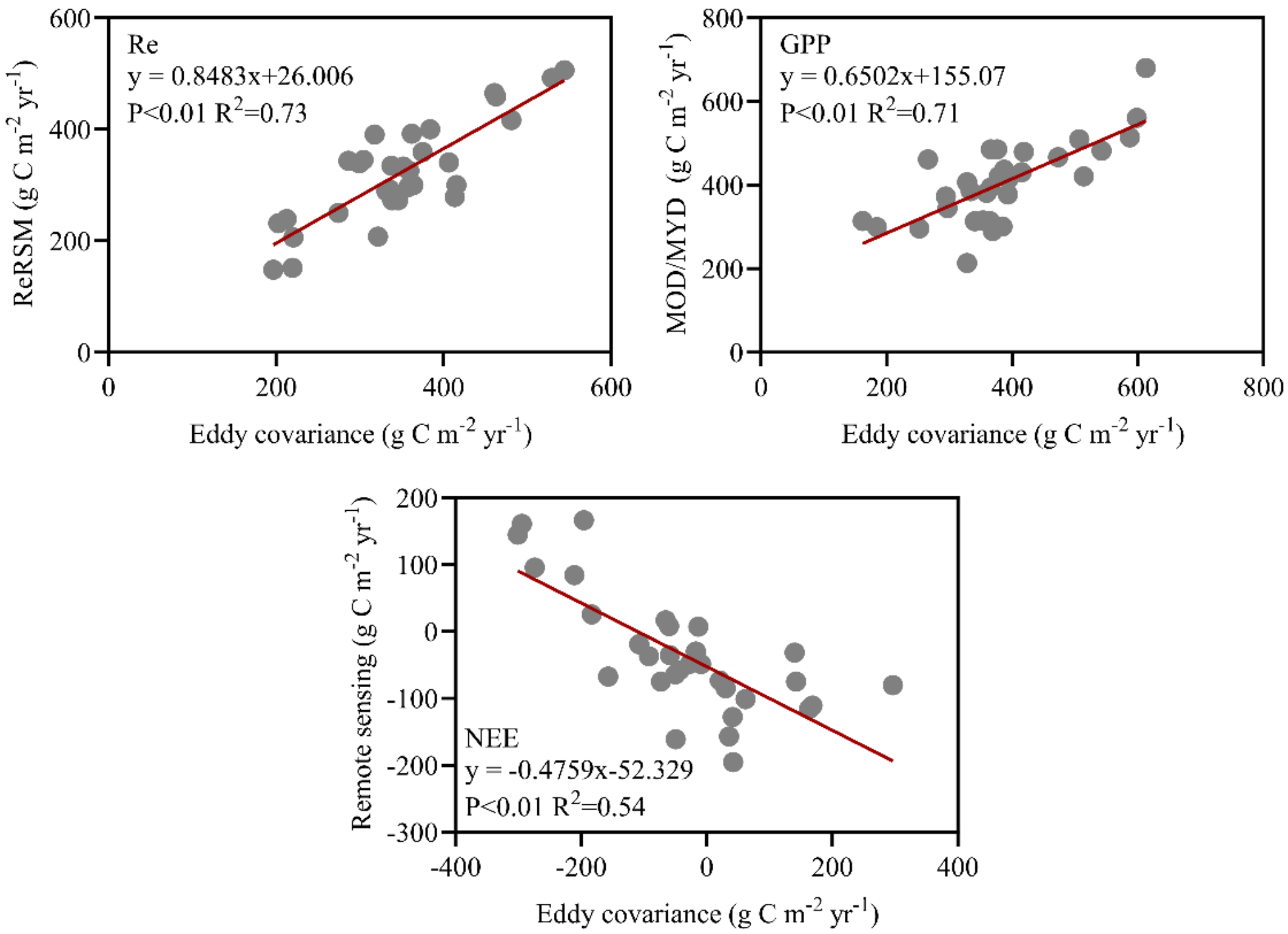

Figure S7 Comparison of remote sensing data and open-path eddy covariance data based site-scale. **Related to Figure 7 and Figure 8.**

Table S3 Spearman rank correlation for different OPEC sites. **Related to Figure 6.**

**Site**	**Ecosystem type**	**Effect**	**Rst.**	**P**
YK	Meadow	Ta	0.56	0.018
		Pre	0.64	0.005
		Ms	0.28	0.23
		Ts	0.38	0.04
		NDVI	0.66	0.008
HBM	Meadow	Ta	0.41	0.03
		Pre	0.58	0.013
		Ms	0.36	0.07
		Ts	0.40	0.04
		NDVI	0.35	0.06
DSL	Swamp meadow	Ta	0.49	0.03
		Pre	0.61	0.01
		Ms	0.52	0.031
		Ts	0.13	0.04
		NDVI	0.37	0.05
AR	Meadow	Ta	0.43	0.03
		Pre	0.72	0.003
		Ms	0.13	0.39
		Ts	0.36	0.04
		NDVI	0.68	0.03
HS	Shrub	Ta	-0.28	0.096
		Pre	0.70	0.002
		Ms	0.62	0.016
		Ts	-0.32	0.05
		NDVI	0.33	0.05

Note: Rst. represents the coefficient of Spearman rank correlation, the closer the Rst. is to 1 or -1, the stronger the positive monotonicity or negative monotonicity. The closer the coefficient is to 0, the less monotonicity exists.

Table S4 The impact of variations in temperature on NEP (mean ± SD). **Related to Figure 5.**

	**Ta increasing**	**Ta decreasing**	**Ts increasing**	**Ts decreasing**
N	19	19	16	23
V_T (℃)	0.61±0.52	-0.61±0.53	0.56±0.41	-0.58±0.53
V_NEP (g C/m^2 yr)	-0.04±55.4	13.7±27.2	-0.05±54.6	5.07±39.7

Note: N represents the sample size, V-T represents the variations in temperature, and V-NEP represents the variations in net ecosystem productivity.

Table S5 The impact of variations in precipitation on NEP (mean ± SD). **Related to Figure 5.**

	**Pre increasing**	**Pre decreasing**
N	22	18
V_Pre (mm)	35.4±25.6	-46.4±38.3
V_NEP (g C/m^2 yr)	33.5±51.3	-25.0±43.5

Note: N represents the sample size, V-Pre represents the variations in temperature, and V-NEP represents the variations in net ecosystem productivity.

Table S6 Comparison between OPEC data of Qilian Mountains and other ecosystems. **Related to Figure 9.**

Site ID	Longitude	Latitude	Elevation (m)	Period	Ecosystem	NEP (g C m^{-2} yr^{-1})	Source
HBM	101.38°E	37.75°N	3250	2015-2020	Meadow	358.7	This study
YK	100.24°E	38.01°N	4148	2016-2022	Meadow	184.2	This study
DSL	98.94°E	38.84°N	3739	2016-2022	Meadow	523.5	This study
MQ	74.95°E	38.66°N	3668	2016	Meadow	64.3	Wei et al., 2021[3]
LB	100.56°E	34.27°N	3980	2008	Meadow	103	Wang et al., 2017[4]
QHM	98.59°E	37.70°N	3718	2018-2019	Meadow	183.6	Liu et al., 2024[5]
NASDE	102.87°E	33.93°N	4270	2009	Peatland	62.7	Wang et al., 2021[6]
NAMORS	102.65°E	33.10°N	4730	2013-2014	Peatland	-15.1	Wang et al., 2021[6]
AR	100.46°E	38.04°N	3033	2016-2022	Meadow	288.5	This study
HS	101.21°E	37.48°N	3180	2011-2020	Shrub	238.6	This study
SZZ	88.7°E	30.95°N	4750	2016-2020	Peatland	155.1	Wei et al., 2021[3]
REG	102.33°E	33.74°N	3423	2008-2010	Wetland	202.1	Kang et al., 2014[7] Wang et al., 2016[8]
XL lake	100.78°E	36.65°N	3214	2012	Lake	314.4	Wang, 2015[9]
HY	100.85°E	36.95°N	3140	2010-2011	Meadow	73.9	Zhang et al., 2012[10]
SL	98.32°E	38.42°N	3885	2009-2011	Meadow	131	Wu et al., 2013[11]
BJ	92.01°E	31.64°N	4410	2014	Meadow	-1.51	Wang et al., 2019[12]
Tdg	101.42°E	36.55°N	3200	2005	Peatland	49.2	Zhang et al., 2008[13]

Mgh	90.3°E	31.42°N	4700	2014-2015	Grassland	-10.2	Wang et al., 2018[14]
HBPF	101.3°E	37.6°N	3250	2003-2004	Shrub	67	Zhao et al., 2006[15]

Reference

1. Lafleur, P., Roulet, N., & Admiral, S. (2001). Annual cycle of CO_2 exchange at a bog peatland. *J. Geophys. Res. Atmos*. 106, 3071–3081, https://doi.org/ 10.1029/2000JD900588.

2. Song, C.C., Sun, L., Huang, Y., Wang, Y.S., & Wan, Z.M. (2011). Carbon exchange in a freshwater marsh in the Sanjiang Plain, northeastern China. *Agric. For. Meteorol.* 151 (8), 1131–1138. https://doi.org/10.1016/j.agrformet.2011.04.001.

3. Wei, D., Qi, Y.H., Ma, Y.M., et al., 2021. Plant uptake of CO_2 outpaces losses from permafrost and plant respiration on the Tibetan Plateau. *Proc Natl Acad Sci USA*. 118(33), e201528311. https://doi.org/10.1073/pnas.2015283118.

4. Wang, B., Jin, H.Y., Chen, D.D., et al., 2017. Diurnal and Seasonal Variations in the Net Ecosystem CO2 Exchange of a Pasture in the Three-River Source Region of the Qinghai−Tibetan Plateau. *PLoS ONE* 12(1): e0170963. https://doi.org/10.1371/journal.pone.0170963.

5. Liu, Y.W., Chen, R.S., Han, C.T., et al., 2024. Research on the characteristics, driving mechanism and spatial pattern of carbon sink in alpine ecosystem: A study case of Qilian Mountains. *Agric. For. Meteorol.* 356, 110166. https://doi.org/10.1016/j.agrformet.2024.110166.

6. Wang, Y.Y., Xiao, J.F., Mao, Y.M., et al., 2021. Carbon fluxes and environmental controls across different alpine grassland types on the Tibetan Plateau. *Agric. For. Meteorol.* 311, 108694. https://doi.org/10.1016/j.agrformet.2021.108694.

7. Kang, X.M., Wang, Y.F., Chen, H., et al., 2014. Modeling Carbon Fluxes Using Multi-Temporal MODIS Imagery and CO2 Eddy Flux Tower Data in Zoige Alpine Wetland, South-West China. *Wetlands* 34(3), 603-618. https://doi.org/10.1007/s13157-014-0529-y

8. Wang, S.Y., Zhang, Y., Lü, S.H., et al., 2016. Biophysical regulation of carbon fluxes over an alpine meadow ecosystem in the eastern Tibetan Plateau. *Int. J. Biometeorol*. 60, 801-812. https://doi.org/10.1007/s00484-015-1074-y.

9. Wang, J.M., 2015. Qinghai lack alpine wetland ecosystem CO_2 exchange characters and it's impact factors research. Thesis, Qinghai Normal University, Xining. (In Chinese with English abstract)

10. Zhang, F.W., Li, Y.N., Cao, G.M., et al., 2012. CO_2 fluxes and their driving factors over alpine meadow grassland ecosystems in the northern shore of Qinghai Lake, China. *Chinese Journal of Plant Ecology* 36, 187-198. (In Chinese with English abstract)

11. Wu, H., Ye, B.S., Wu, J.K., et al., Observations and study on the CO2 flux in an alpine meadow ecosystem in the upper reaches of the Shule River Basin. *Acta Prataculturae Sinica* 21-29. (in Chinese with English Abstract).

12. Wang, Q.F., Jin, H.J., Wen, J., et al., 2019. Hydro-meteorological influences on the growing season CO_2 exchange of an alpine meadow in the northeastern Tibetan Plateau permafrost region: observations using eddy covariance method. *Theor. Appl. Climatol.* 138, 1063–1073. https://doi.org/10.1007/s00704-019-02861-5.

13. Zhang, F.W., Liu, A.H., Li, Y.M., et al., 2008. CO_2 flux in alpine wetland ecosystem on the Qinghai-Tibetan Plateau, China. *Acta Ecologica Sinica* 28, 453-462. (In Chinese with English Abstract)

14. Wang, L., Liu, H., Shao, Y.P., et al., 2018. Water and CO_2 fluxes over semiarid alpine steppe and humid alpine meadow ecosystems on the Tibetan Plateau. *Theor. Appl. Climatol.* 131, 547-556. https://doi.org/10.1007/s00704-016-1997-1.

15. Zhao, L., Li, Y.N., Xu, S.X., Zhou, H.K., et al., 2006. Diurnal, seasonal and annual variation in net ecosystem CO2 exchange of an alpine shrubland on Qinghai-Tibetan plateau. *Glob. Change Biol.* 12, 1940-1953. https://doi.org/10.1111/j.1365-2486.2006.01197.x.
